# Cost-effectiveness of financial incentives and disincentives for improving food purchases and health through the US Supplemental Nutrition Assistance Program (SNAP): A microsimulation study

**DOI:** 10.1371/journal.pmed.1002661

**Published:** 2018-10-02

**Authors:** Dariush Mozaffarian, Junxiu Liu, Stephen Sy, Yue Huang, Colin Rehm, Yujin Lee, Parke Wilde, Shafika Abrahams-Gessel, Thiago de Souza Veiga Jardim, Tom Gaziano, Renata Micha

**Affiliations:** 1 Friedman School of Nutrition Science and Policy, Tufts University, Boston, Massachusetts, United States of America; 2 Harvard T.H. Chan School of Public Health, Boston, Massachusetts, United States of America; 3 Department of Epidemiology and Population Health, Albert Einstein College of Medicine, Bronx, New York, United States of America; Stanford University, UNITED STATES

## Abstract

**Background:**

The Supplemental Nutrition Assistance Program (SNAP) provides approximately US$70 billion annually to support food purchases by low-income households, supporting approximately 1 in 7 Americans. In the 2018 Farm Bill, potential SNAP revisions to improve diets and health could include financial incentives, disincentives, or restrictions for certain foods. However, the overall and comparative impacts on health outcomes and costs are not established. We aimed to estimate the health impact, program and healthcare costs, and cost-effectiveness of food incentives, disincentives, or restrictions in SNAP.

**Methods and findings:**

We used a validated microsimulation model (CVD-PREDICT), populated with national data on adult SNAP participants from the National Health and Nutrition Examination Survey (NHANES) 2009–2014, policy effects from SNAP pilots and food pricing meta-analyses, diet–disease effects from meta-analyses, and policy, food, and healthcare costs from published literature to estimate the overall and comparative impacts of 3 dietary policy interventions: (1) a 30% incentive for fruits and vegetables (F&V), (2) a 30% F&V incentive with a restriction of sugar-sweetened beverages (SSBs), and (3) a broader incentive/disincentive program for multiple foods that also preserves choice (SNAP-plus), combining 30% incentives for F&V, nuts, whole grains, fish, and plant-based oils and 30% disincentives for SSBs, junk food, and processed meats. Among approximately 14.5 million adults on SNAP at baseline with mean age 52 years, our simulation estimates that the F&V incentive over 5 years would prevent 38,782 cardiovascular disease (CVD) events, gain 18,928 quality-adjusted life years (QALYs), and save $1.21 billion in healthcare costs. Adding SSB restriction increased gains to 93,933 CVD events prevented, 45,864 QALYs gained, and $4.33 billion saved. For SNAP-plus, corresponding gains were 116,875 CVD events prevented, 56,056 QALYs gained, and $5.28 billion saved. Over a lifetime, the F&V incentive would prevent approximately 303,900 CVD events, gain 649,000 QALYs, and save $6.77 billion in healthcare costs. Adding SSB restriction increased gains to approximately 797,900 CVD events prevented, 2.11 million QALYs gained, and $39.16 billion in healthcare costs saved. For SNAP-plus, corresponding gains were approximately 940,000 CVD events prevented, 2.47 million QALYs gained, and $41.93 billion saved. From a societal perspective (including programmatic costs but excluding food subsidy costs as an intra-societal transfer), all 3 scenarios were cost-saving. From a government affordability perspective (i.e., incorporating food subsidy costs, including for children and young adults for whom no health gains were modeled), the F&V incentive was of low cost-effectiveness at 5 years (incremental cost-effectiveness ratio: $548,053/QALY) but achieved cost-effectiveness ($66,525/QALY) over a lifetime. Adding SSB restriction, the intervention was cost-effective at 10 years ($68,857/QALY) and very cost-effective at 20 years ($26,435/QALY) and over a lifetime ($5,216/QALY). The combined incentive/disincentive program produced the largest health gains and reduced both healthcare and food costs, with net cost-savings of $10.16 billion at 5 years and $63.33 billion over a lifetime. Results were consistent in probabilistic sensitivity analyses: for example, from a societal perspective, 1,000 of 1,000 iterations (100%) were cost-saving for all 3 interventions. Due to the nature of simulation studies, the findings cannot prove the health and cost impacts of national SNAP interventions.

**Conclusions:**

Leveraging healthier eating through SNAP could generate substantial health benefits and be cost-effective or cost-saving. A combined food incentive/disincentive program appears most effective and may be most attractive to policy-makers.

## Abstract 2

In a microsimulation study Dariush Mozaffarian and colleagues model 3 different potential food choice interventions for the benefits of health outcomes and costs-savings.

## Author summary

**Why was this study done?:**

- The US Supplemental Nutrition Assistance Program (SNAP) provides approximately $70 billion/year for food purchases for low-income households, supporting about 1 in 7 Americans.
- Diet-related diseases and costs disproportionately harm low-income individuals, making SNAP a major potential lever to improve food choices, reduce healthcare costs, and improve disparities.
- However, the overall and comparative impacts and cost-effectiveness of food incentives, disincentives, and restrictions in SNAP for health outcomes and costs are not established.

**What did the researchers do and find?:**

- Using nationally representative data and a validated microsimulation model, we evaluated 3 potential interventions within SNAP, including (1) a 30% subsidy for purchases of fruits and vegetables (F&V) (F&V incentive), (2) a F&V incentive with restriction of sugar-sweetened beverages (SSBs) (F&V incentive/SSB restriction), and (3) a broader incentive/disincentive program that preserves choice (SNAP-plus), combining a 30% subsidy for purchases of F&V, nuts, whole grains, fish, and plant-based oils and a 30% disincentive for purchases of SSBs, junk food, and processed meats.
- Over a lifetime, we found that the F&V incentive, F&V incentive/SSB restriction, and SNAP-plus programs would prevent respectively about 304,000, 798,000, and 940,000 CVD events and −1,000, 171,000, and 147,000 cases of diabetes, with respective healthcare cost-savings of about $7 billion, $39 billion, and $429 billion. From a societal perspective, all interventions were cost-saving. From a government affordability perspective (incorporating food subsidy costs for all 42 million participants in SNAP, including children), the F&V incentive was cost-effective (lifetime incremental cost-effectiveness ratio [ICER] approximately $66,500 per quality-adjusted life year), the F&V incentive/SSB restriction policy was very cost-effective (ICER approximately $5,200), and the SNAP-plus program was cost-saving (saving $63 billion).

**What do these findings mean?:**

- Leveraging healthier eating through SNAP could generate substantial health benefits among low-income Americans and be cost-effective or cost-saving.
- A combined food incentive/disincentive program produced the largest modeled gains in health and healthcare savings.
- These findings support the need to test these SNAP interventions in state pilot programs.

## Introduction

Suboptimal diet is a major cause of poor health, particularly related to cardiometabolic diseases such as coronary heart disease (CHD), stroke, type 2 diabetes, and obesity [1,2]. Nearly half of all cardiovascular and diabetes deaths in the US are linked to poor diet, corresponding to almost 1,000 deaths daily [3]. These adverse health conditions produce tremendous economic burdens [2,4], including for government programs such as Medicare and Medicaid, private health insurers, and self-insured businesses, and for individuals in the form of personal illness, out-of-pocket costs, and lost productivity. The direct and indirect costs of cardiovascular diseases (CVDs) are estimated at $317 billion/year, including $193 billion/year in direct healthcare expenditures and $124 billion/year in lost productivity, while the partly overlapping corresponding costs of type 2 diabetes are $320, $112, and $208 billion/year, respectively. The combined healthcare costs of all obesity-related conditions are estimated at $1.42 trillion/year, or about 8% of US gross domestic product. Further, these diet-related diseases and costs disproportionately harm low-income individuals [4], contributing to major disparities. Clearly, new cost-effective interventions are needed to address these burdens.

The US Supplemental Nutrition Assistance Program (SNAP, formerly the Food Stamp Program) is among the largest and most promising opportunities to improve food choices and health while reducing costs and disparities. Established by the Food Stamp Act in 1964 “to safeguard the health and well-being of the Nation’s population and raise levels of nutrition among low-income households” [5], SNAP provides monthly benefits for retail food purchases to approximately 42 million low-income individuals, or approximately 1 in 7 Americans [6,7]. While SNAP is an essential safety net that successfully reduces food insecurity [8], relatively little programmatic emphasis has been on nutrition or health. Disparities remain or have worsened in the diets of low-income Americans [9–12], and SNAP participants experience significantly higher all-cause, cardiovascular, and diabetes mortality compared to other American adults [13–15]. The size, scope, and potential for impact of SNAP are staggering, with an annual budget of $68 billion/year that exceeds those of the National Institutes of Health ($33 billion), Centers for Disease Control and Prevention ($7 billion), and Food and Drug Administration ($5 billion) combined [7].

SNAP is reauthorized by Congress every 5 years as part of the Farm Bill, due this year. With its size and reach, the 2018 Farm Bill represents one of the most timely and compelling opportunities to improve the nation’s health. Tremendous current interest exists in potential ways to further leverage SNAP to not only address food insecurity but also improve nutrition, health, equity, and health costs [16–23]. For example, a new bipartisan Food Is Medicine Working Group was recently launched in the US Congress to create innovative policies, including within SNAP, to leverage food policy to improve health and to lower healthcare costs [16]. Separately, the influential Bipartisan Policy Center recently created a new SNAP Task Force, co-chaired by former Senator Bill Frist and former US Department of Agriculture (USDA) Secretaries Dan Glickman and Ann Veneman, to provide policy recommendations for using SNAP and related programs to improve health, combat poor nutrition, and reduce healthcare costs [17]. Previously proposed revisions for SNAP have included incorporating financial incentives for purchasing fruits, vegetables, or other healthful foods, and restricting unhealthy items such as sugar-sweetened beverages (SSBs) [18–23]. The USDA Healthy Incentives Pilot (HIP), a randomized controlled trial implemented among SNAP participants, demonstrated that a 30% subsidy for fruits and vegetables (F&V) increased intake by approximately 26% [24]. Incentives for healthful foods could also be combined with partial disincentives on less healthful items, rather than absolute restrictions, preserving choice for participants. Yet, whereas economic incentives and disincentives are known to be effective in changing behavior [24–27], the overall and comparative impacts of these different strategies on health outcomes, program and healthcare costs, and cost-effectiveness are not established, limiting policy decision-making.

Policy-makers, practitioners, and the public are concerned about potential federal changes to programs that aid Americans, requiring new research and data to support how SNAP benefits may be strengthened and leveraged in a way that benefits participants and appeals to lawmakers. Understanding not only health impacts, but also costs and cost-effectiveness, is a crucial tool for such conversations. To elucidate the effects of different strategies to improve SNAP and provide evidence to help inform SNAP policy, a validated microsimulation model was used to estimate the potential health impact of a F&V incentive, a F&V incentive plus SSB restriction, and a combined incentive/disincentive program on multiple foods within SNAP on cardiometabolic outcomes. Furthermore, the corresponding programmatic and healthcare costs and comparative cost-effectiveness of these different strategies were evaluated. This work was performed as part of the Food Policy Review and Intervention Cost-Effectiveness (Food-PRICE) project (https://www.food-price.org/).

## Methods

### Study overview

**Fig 1:**
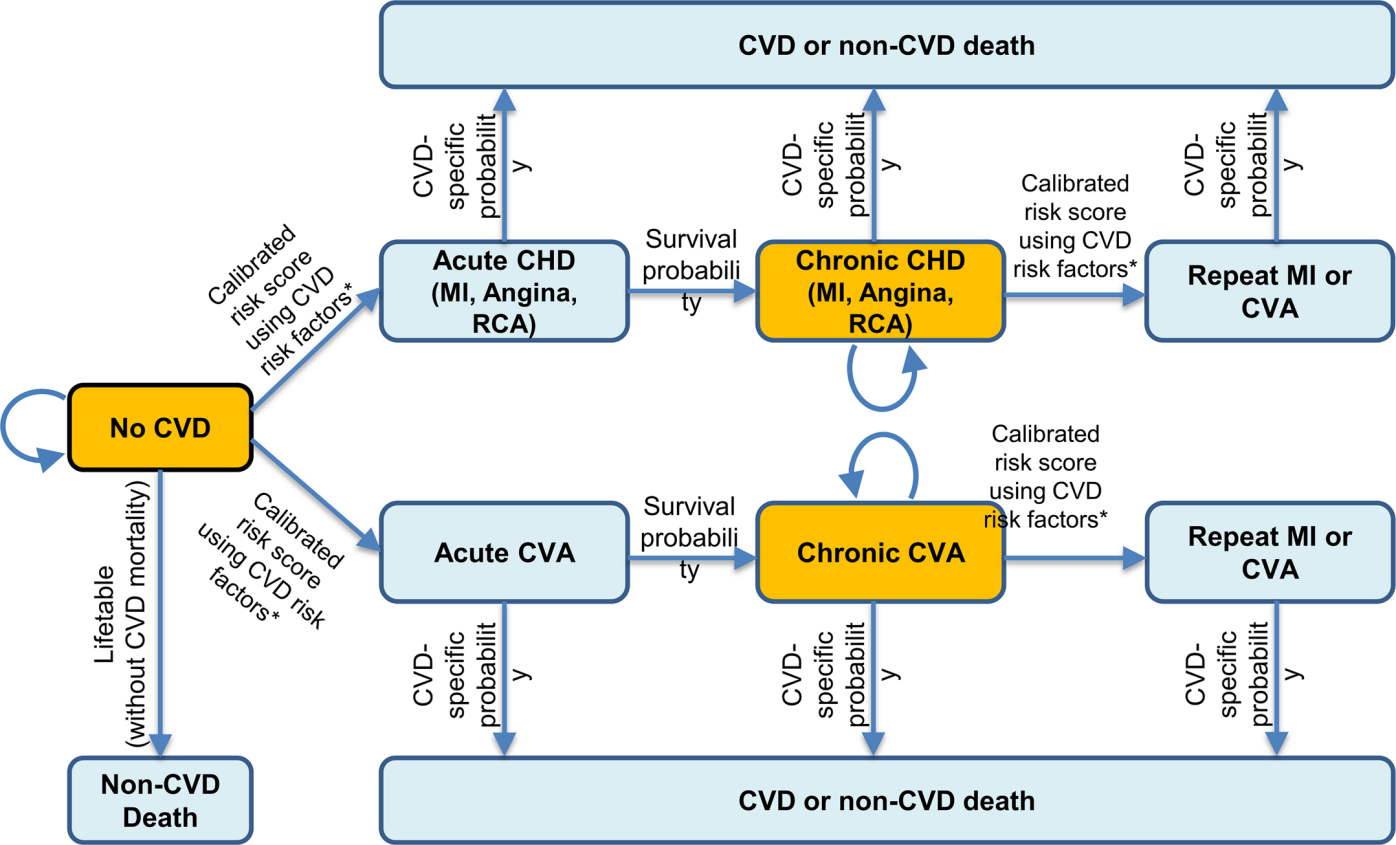
The CVD-PREDICT microsimulation model. Transitions were based on a calibrated risk score including age, sex, systolic blood pressure, total cholesterol, HDL-cholesterol, current smoking, and diabetes status. Diabetes outcome was also simulated as a model event. *Baseline risk factors were derived from NHANES 2009–2014, with further annual changes in risk factors incorporating both age and secular trends. MI, myocardial infarction; CHD, coronary heart disease; CVA, cerebrovascular accident; CVD, cardiovascular disease; HDL-cholesterol, high-density lipoprotein cholesterol; NHANES, National Health and Nutrition Examination Survey; RCA, resuscitated cardiac arrest.

The potential health and economic impacts of implementing specific food incentive, restriction, or disincentive programs within SNAP were modeled using the validated CVD-PREDICT microsimulation model [28,29] over a 5-, 10-, and 20-year analytic period (2018–2038) as well as over the cohort lifetime. Populated with individuals from the National Health and Nutrition Examination Survey (NHANES), the CVD-PREDICT model utilized national data on baseline characteristics, risks, dietary habits, and disease incidence to assess cumulative cardiometabolic health outcomes and costs based on current trends as well as alternative scenarios of specific interventions (Fig 1; S1 Text). At each stage of the logic pathway, the best available evidence, supplemented with reasoned assumptions, was used to estimate the potential health and economic effects of the respective food price policies implemented among low-income Americans participating in SNAP. The elements of the model inputs, structure, and outputs are each described in further detail below. This investigation utilized de-identified datasets and was exempt from human subjects approval.

### SNAP intervention scenarios

We modeled 3 distinct interventions within SNAP, including (1) a 30% financial incentive for purchases of F&V (F&V incentive), (2) a 30% F&V incentive with restriction of SSBs (F&V incentive/SSB restriction), and (3) a broader incentive/disincentive program that preserves choice (SNAP-plus), combining a 30% financial incentive for purchases of F&V, nuts, whole grains, fish, and plant-based oils and a 30% disincentive for purchases of SSBs, junk food, and processed meats (see S1 Table for food category details and definitions). These 3 interventions were predefined based on current policy evidence and interest in the US [17–24,27,30] (F&V incentives and SSB restrictions) and worksite wellness and insurance benefit programs that incentivize a broad range of healthy foods (SNAP-plus) [31,32], focusing on foods with the largest estimated cardiometabolic impact in the US [3]. We performed a secondary analysis of SNAP-plus excluding incentives for fish and plant-based oils, the 2 most expensive categories.

We modeled the implementation of each intervention through use of the existing SNAP electronic benefits transfer (EBT) card, which can be used like a debit card at diverse retail outlets including supermarkets, grocery stores, convenience stores, and farmers markets and is linked to product-identifying Universal Product Codes (UPCs). Use of EBT takes advantage of this established system with existing product-specific inclusions and restrictions (e.g., purchases of alcohol and preprepared hot foods are excluded), permitting technology-based financial nudges. In SNAP-plus, for example, for every SNAP dollar spent on incentivized foods, $0.30 would be returned to the EBT account (producing a net additional expense to SNAP), while for every SNAP dollar spent on disincentivized foods, $1.30 would be debited from the EBT card (producing a net relative savings to SNAP that could help offset the cost of incentives). Each intervention was accompanied by modeled coordinated outreach and education to SNAP participants, accounted for in policy effect sizes and implementation and ongoing annual costs (see below).

### Simulated US population

Our population was based on Americans aged 35–80 years at baseline across 3 cycles of NHANES (2009–2010, 2011–2012, 2013–2014) who participated in SNAP, defined by reporting household SNAP benefits in the past 12 months (Table 1). The sociodemographic characteristics, cardiometabolic risk factors, and dietary habits of these participants were derived from NHANES data, including dietary habits from two 24-hour recalls per person as previously described [9]. Utilizing NHANES survey weights to account for the complex survey design and sampling, we sampled the study population with replacement to create a simulated nationally representative SNAP population of 1,000,000 individuals, who were followed until death or age 100, whichever came first. We recognized that individuals may enter and leave SNAP over time, and considered this impact as further described in S2 Text. Data from NHANES are publicly available at https://www.cdc.gov/nchs/nhanes/; other data inputs are from published papers or published in the present report.

**Table 1:** Sociodemographic characteristics of US adults aged 35–80 years at baseline participating in the Supplemental Nutrition Assistance Program (SNAP)^a^.

Characteristic	Value^b^
**Number of US adults represented (age 35–80 y)**	14.5 million^c^
**Age in years, mean (SD)**	52.1 (12.3)
**Age in years, percent**	
35–44	29.4
45–54	30.9
55–64	24.7
65–74	10.6
75–80	4.4
**Sex, percent**	
Male	45.7
Female	54.3
**Race/ethnicity, percent**	
Non-Hispanic white	50.8
Non-Hispanic black	24.3
Hispanic	18.9
Other	6.0
**Education level, percent**	
Less than high school graduate	36.1
High school graduate^d^	28.9
Some college	28.0
College graduate	7.0
**Family income to poverty ratio, percent**^**e**^	
<1.30	67.4
1.30–1.84	13.9
1.85–2.99	11.2
≥3.00	7.5
**Federal health insurance, percent**^**f**^	
Medicare	22.4
Medicaid	29.5
Dual-eligible	10.2
Medicare, Medicaid, or both	41.7

^a^The modeled sample was drawn from all SNAP participants in combined cycles of the 2009–2014 National Health and Nutrition Examination Survey (NHANES), incorporating NHANES sampling and survey weights. Demographics and other participant and family characteristics are therefore representative of these NHANES-sampled SNAP participants, with a survey sample size of 1,941 adult SNAP participants aged 35–80 years with two 24-hour dietary recalls and reporting receiving household SNAP benefits in the past 12 months.

^b^Value represents mean (SD) for continuous variables and percent for categorical variables. Percentages may not sum to 100% due to rounding.

^c^Based on the total number of SNAP participants in 2017 (42,138,000) [7] and the percentage of adult SNAP participants (aged 35+) in 2016 (34.5%) [33].

^d^Including general equivalency diploma (GED).

^e^The ratio of family income to the federal poverty threshold, adjusting for household size. For reference, the 2014 federal poverty threshold for a family of 4 was $23,850/year.

^f^Dual-eligible beneficiaries are on both Medicare and Medicaid. Based on NHANES 2009–2014, among adults aged 35+ years, 61.1% of those on Medicaid are also on SNAP, while 12.7% of those on Medicare are also on SNAP.

### Policy effects on dietary habits

Details of the sources for policy effects and corresponding calculations are provided in S2 Table. Estimated policy effects accounted for the expected change in intake of each food item for a 30% price change, the percent of overall food purchased using SNAP dollars, the percent of each food item purchased at SNAP venues (e.g., supermarkets, grocery stores, and farmers markets, as opposed to restaurants and food banks), and the expected shifting in food spending from SNAP to other food dollars as a result of the intervention. Consistent with empirical results of interventions that demonstrate rapid changes in consumption with price changes [24–26,34], we did not model any time lag between onset of the intervention and dietary change.

The effects of the F&V incentive were derived from HIP, a randomized trial in SNAP participants that provided a 30% subsidy for F&V purchases [24,25]. This effect size incorporated the average coverage and participant response to the program, its corresponding outreach and education, and potential shifts in spending between SNAP versus non-SNAP dollars (S3 Text). For the combined incentive/disincentive program, this effect was extended to the other incentivized food groups, as this provided a more conservative effect size than larger estimated effects of price subsidies on healthful food items from a recent meta-analysis of interventional and prospective studies [26]. While SSB restriction would prohibit purchases using SNAP dollars, we recognized and accounted for the likely average response of individuals who might shift some of their spending on SSBs from SNAP dollars to other food dollars, as noted above. Accounting for each of these data sources and considerations, we estimated that a 30% F&V incentive in SNAP would increase overall intake of fruits by 23.4% and vegetables by 19.0%; an SSB restriction in SNAP would reduce overall intake of SSBs by 33.2%; a 30% incentive in SNAP for other healthful foods would increase their overall intake by between 19.0% and 24.2%; and a 30% disincentive in SNAP for SSBs, junk food, and processed meats would decrease their overall intake by between 13.3% and 17.4% (S2 Table). S2 Table also describes several sensitivity analyses for these assumptions and corresponding calculations.

### Effects of dietary changes on cardiometabolic risk

Our detailed methods for reviewing and synthesizing evidence to estimate effect sizes for associations between dietary factors and cardiometabolic endpoints have been reported [1,3]. We utilized evidence from meta-analyses of prospective cohorts or randomized clinical trials evaluating direct associations of dietary factors with CHD, stroke, or type 2 diabetes, by age; associations of SSBs and added sugar from other foods with body mass index (BMI) and additional BMI-independent associations of SSBs with CHD, stroke, and diabetes, by age and overweight/obesity status; and effects of dietary sodium on blood pressure, by age, race, and hypertensive status (see S3 Table). To estimate cardiometabolic effects of junk food, we assessed the independent relationship between US junk food consumption and 13 other dietary components with established links to cardiometabolic risk (S4 Table), such as fruits, vegetables, whole grains, nuts, omega-3 fats, potassium, red meat, processed meat, polyunsaturated fat as a replacement for carbohydrate or saturated fat, and SSBs. Using the identified 9 joint associations, some of which predicted harm and some of which predicted benefit, we estimated the etiologic effect of changes in junk food intake on cardiometabolic risk.

These estimated effects can model associations with cardiometabolic risk if there is not substantial bias from confounding (which might overestimate effects) or measurement error (which might underestimate effects). To reduce bias from confounding, all identified observational studies in the utilized meta-analyses included multivariable adjustment for other risk factors. Measurement error was generally not addressed, although some studies included serial dietary measures. We also recognized that associations of individual dietary components with health may differ from joint associations when foods are consumed as diet patterns: for example, due to clustering of healthful components such as fruits, vegetables, and whole grains, or unhealthful components such as SSBs and processed meats. We performed detailed validity analyses evaluating the extent to which estimated etiologic effects might be biased due to these limitations [1], including comparing estimated etiologic effects for individual dietary components to (1) observed associations of overall dietary patterns with clinical endpoints in long-term cohorts, (2) effects of dietary patterns on low-density lipoprotein cholesterol (LDL-cholesterol) and systolic blood pressure in randomized controlled feeding trials, and (3) effects of dietary patterns on hard CVD endpoints in a large randomized clinical trial (S4 Text; S11–S13 Tables). Each of these validity analyses demonstrated that estimated etiologic effects for individual dietary components were very similar to what would be expected based on these other lines of evidence [1]. These observed etiologic effects also inherently account for the average dietary complements and substitutes in the population, as further described in S4 Text.

### Microsimulation model structure and outputs

CVD-PREDICT is a validated discrete time microsimulation dynamic model, coded in C++, that simulates and quantifies the effects of policies on cardiometabolic outcomes including CHD, stroke, and type 2 diabetes, with annual updating of each health state (S1 Text) [28,29]. The model is run at the level of individuals, incorporating the probability of their health transitions according to each person’s risk factors. Based on NHANES as described above, the model was populated with simulated SNAP participants and their corresponding risk factors including data on age, sex, systolic blood pressure, total cholesterol, HDL-cholesterol, BMI, smoking status, diabetes status, baseline dietary habits, and other characteristics as necessary. Each individual’s risk was calculated annually, adapted primarily from the Framingham Risk Score, with calibration and validation to empirical historical disease trends [29] and with modeled transition health states including disease-free, death, and acute or chronic CHD events, stroke (cerebrovascular accident), and diabetes. CVD risk factors and subsequent estimated CVD incidence and mortality were extrapolated and updated using age and time trends from NHANES. At any given time point, a simulated individual could only be in 1 health state, with the probability of experiencing subsequent events based on individual characteristics and risk factors. The microsimulation process across each state and transitions are illustrated in Fig 1.

Model outputs included total and mean estimated outcomes for the simulated population over a 5-, 10-, and 20-year open cohort analytic period (2018–2038) as well as a closed cohort lifetime (S1 Text). The specific model outcomes included deaths from CHD or stroke, nonfatal events including myocardial infarction, stroke, angina, resuscitated cardiac arrest, and type 2 diabetes, quality-adjusted life years (QALYs), and event-associated healthcare costs (see below).

### Intervention and healthcare costs

Intervention costs were estimated including administrative costs of program implementation, EBT processing, retail infrastructure, outreach and education, and monitoring and evaluation; SNAP program subsidy costs for incentivized foods; and SNAP program savings for disincentivized foods (but not restricted foods, which produced no program savings) (S5 Table). Administrative costs were derived from the HIP report estimates for extending the project nationally [25]. Because SNAP is an established national program with benefits operationalized through an existing electronic system at retailers that already discriminates products based on UPCs, retailers were considered unlikely to have any new meaningful costs due to implementation of the policies. The costs of food incentives and disincentives were calculated for each food category based on data from the USDA Economic Research Service Quarterly Food-at-Home Price Database [35], the estimated average national price of SSBs [36], the Consumer Expenditure Survey [37], and the USDA SNAP report [38].

As reported in detail elsewhere [29], direct healthcare costs were derived from various sources for all acute and chronic disease states, surgical procedures, screening, treatments, and side effects (S6 Table). The total annual costs for individuals in each disease state were calculated using the weighted average of nonfatal and fatal states. The costs associated with chronic disease states were derived from various studies on costs for each individual disease state. Indirect costs such as lost productivity were conservatively excluded from the analysis.

### Cost-effectiveness analysis

In accordance with recommendations from the Second Panel on Cost-Effectiveness in Health and Medicine [39], we conducted analyses from various perspectives. This included (1) a societal perspective, incorporating program administrative costs and healthcare costs but not costs or savings from direct food subsidies or disincentives (which can be considered an intra-societal cash transfer); (2) a government affordability perspective further incorporating SNAP program costs and savings from direct food subsidies and disincentives for all SNAP adults (age 35+ y); and (3) a government affordability perspective further incorporating SNAP program costs and savings from direct food subsidies and disincentives for all SNAP participants (half of whom are children, for whom our model estimated only costs, with no health benefits or healthcare cost-savings). Each government affordability perspective can also be considered as a modified societal perspective that includes predominantly government healthcare and SNAP costs, while also adding the considerable government costs of food subsidies—an intra-societal transfer to the public not typically included in a societal perspective—and also intervention and health costs borne by other health payers and consumers.

All costs were inflated to constant 2017 US dollars, and all costs and health endpoints discounted annually by 3%. Net costs were calculated as intervention costs minus healthcare cost-savings from reduced cardiometabolic diseases. Incremental cost-effectiveness ratios (ICERs) were calculated as the net change in costs divided by the net change in QALYs. Willingness-to-pay thresholds were evaluated at $150,000 and $50,000 per QALY, consistent with American Heart Association/American College of Cardiology recommendations [40].

### Probabilistic sensitivity analyses

To assess the potential impact of uncertainty in key inputs, we performed probabilistic sensitivity analyses jointly incorporating the uncertainty distributions of multiple parameters. This uncertainty included uncertainty in policy effect sizes, diet–disease relative risks (including their variation by age), CVD risks, implementation costs, food units costs, formal and informal healthcare costs, and utility weights (S9 Table). One thousand simulations were run drawing from the uncertainty distributions of each of these inputs at 5 years and over a lifetime, with 95% uncertainty intervals (UIs) based on the 2.5th and 97.5th percentiles of the 1,000 simulations.

## Results

### Population characteristics and dietary intakes

The mean (SD) age of adult SNAP participants was 52.1 (12.3) years, about half (50.8%) were white, and more than 1 in 3 participants (35.0%) had some college education or more (Table 1). Among adults on SNAP, 22.4% were on Medicare, 29.5% on Medicaid, and 41.7% on either Medicare or Medicaid (with 10.2% on both, i.e., dual-eligible). Considering adults on Medicare or Medicaid as the denominator, 12.7% of Medicare and 61.1% of Medicaid beneficiaries were also on SNAP.

The current baseline consumption levels and estimated changes in consumption with the policy interventions are shown in Table 2. The F&V subsidy would increase intakes of fruits by 19.1 g/d and vegetables by 25.6 g/d. Adding an SSB restriction would lower SSB intake by 139 g/d (4.7 fl oz/d). A combined incentive/disincentive program would have a smaller effect on SSBs (−1.7 fl oz/d), but would also modestly increase intakes of whole grains, fish, and plant-based oils and modestly reduce intakes of processed meats and junk food. To place these changes in perspective as a proportion of the 2015 Dietary Guidelines for Americans recommendations [41] (for a 2,000 kcal/d diet, the following equivalents: fruits, 2 cups/d; vegetables, 2.5 cups/d; nuts, 5 oz/wk; whole grains, 3 oz/d; and fish, 8 oz/wk), the changes in intake with SNAP-plus would achieve 9.6% of the recommendations for fruits, 11.5% for vegetables, 8.5% for nuts, 4.0% for whole grains, and 12.1% for fish. Such comparisons were not made for unhealthy food items like SSBs, where the optimal intake could be considered 0.

**Table 2:** Baseline consumption levels and estimated changes in consumption per person at 5 years for 3 intervention scenarios in SNAP^a^.

Food category	Baseline consumption^b^	Change in consumption^c^		
		F&V incentive	F&V incentive/SSB restriction	SNAP-plus
Fruits, g/d	81.3	19.1	19.1	19.1
Vegetables, g/d	134.8	25.6	25.6	28.8
Nuts, g/d	5.4	—	—	1.7
Whole grains, g/d	13.9	—	—	3.4
Fish, g/d	20.6	—	—	3.9
Plant-based oils, g/d	18.7	—	—	3.8
SSBs, g/d (fl oz/d)	414 (14.0)	—	−139 (−4.7)	−52 (−1.7)
Processed meat, g/d	31.8	—	—	−5.1
Junk food, g/d	66.5	—	—	−11.5

^a^The 3 interventions were (1) a 30% financial incentive for purchases of F&V (F&V incentive), (2) a 30% F&V incentive plus restriction of SSBs (F&V incentive/SSB restriction), and (3) a broader 30% incentive/disincentive program for multiple foods that preserves choice (SNAP-plus) including a 30% financial incentive for purchases of fruits, vegetables, nuts, whole grains, fish, and plant-based oils and a 30% disincentive for purchases of SSBs, junk food, and processed meats (see S1 Table for food category details and definitions).

^b^Derived using data from NHANES 2009–2014, based on adult SNAP participants with two 24-hour dietary recalls per person (see S1 Table).

^c^Estimated policy effects accounted for the expected change in intake of each food item due to the intervention, the percent of overall food purchased using SNAP dollars, the percent of each food item purchased at SNAP venues (e.g., supermarkets, grocery stores, and farmers markets), and expected shifting in food spending from SNAP to other food dollars as a result of the intervention.

F&V, fruits and vegetables; NHANES, National Health and Nutrition Examination Survey; SNAP, Supplemental Nutrition Assistance Program; SSB, sugar-sweetened beverage.

### Health outcomes

Health gains, healthcare cost-savings, intervention costs, and ICERs are summarized in Table 3, with results of probabilistic sensitivity analyses in S10 Table. At 5 years, our simulation estimated that the F&V incentive would prevent 38,782 CVD events, with minimal effects on diabetes; the F&V incentive/SSB restriction policy would prevent 93,933 CVD events and 30,443 cases of diabetes; and SNAP-plus would prevent 116,875 CVD events and 26,138 cases of diabetes. Over a lifetime, corresponding values were 303,911, 797,888, and 939,965 CVD events prevented and −1,077, 171,357, and 146,590 cases of diabetes prevented, with respective healthcare cost-savings of $6.77 billion, $39.16 billion, and $41.93 billion.

**Table 3:** Estimated health gains, costs, and cost-effectiveness of SNAP food subsidies, restrictions, and combined incentives/disincentives over 5, 10, 20 years and over a lifetime^a^.

Outcome	5 years	10 years	20 years	Lifetime
***F&V incentive***				
**Cases averted (95% UI)**				
Total CVD	38,782	71,616	121,788	303,911
Diabetes^b^	−361	56	−203	−1,077
**CVD deaths**	2,926	5,948	11,713	41,394
**QALYs gained**	18,928	59,259	155,792	649,376
**Healthcare cost-savings (dollars, billions)**^**c**^	1.21	2.15	3.48	6.77
**Policy administrative costs (dollars, millions)**	125	144	167	212
**Food subsidy costs (dollars, billions)**				
SNAP adults (age 35+ y)	5.04	8.60	12.99	21.81
All SNAP participants	11.54	19.65	29.66	49.89
**ICER (dollars/QALY), by perspective**^**d**^				
Societal	Saving ($1.16 billion)	Saving ($2.10 billion)	Saving ($3.42 billion)	Saving ($6.69 billion)
Government affordability (subsidizing SNAP adults aged 35+ years)	204,928	109,644	61,451	23,284
Government affordability (subsidizing all SNAP participants)	548,053	296,187	168,455	66,525
***F&V incentive/SSB restriction***^*e*^				
**Cases averted**				
Total CVD	93,933	181,502	333,591	797,888
Diabetes	30,443	65,499	116,993	171,357
**CVD deaths**	9,628	19,517	40,420	130,938
**QALYs gained**	45,864	155,646	457,184	2,106,832
**Healthcare cost-savings (dollars, billions)**^**c**^	4.33	9.00	17.68	39.16
**Policy administrative costs (dollars, millions)**	149	185	230	317
**Food subsidy costs (dollars, billions)**				
SNAP adults (age 35+ years)	5.04	8.60	13.00	21.88
All SNAP participants	11.54	19.66	29.69	50.04
**ICER (dollars/QALY), by perspective**^**d**^				
Societal	Saving ($4.28 billion)	Saving ($8.94 billion)	Saving ($17.60 billion)	Saving ($39.05 billion)
Government affordability (subsidizing SNAP adults aged 35+ years)	16,660	Saving ($0.34 billion)	Saving ($4.60 billion)	Saving ($17.17 billion)
Government affordability (subsidizing all SNAP participants)	158,293	68,857	26,435	5,216
***SNAP-plus (combined incentives/disincentives)***				
**Cases averted**				
Total CVD	116,875	222,355	398,645	939,965
Diabetes	26,138	56,924	99,657	146,590
**CVD deaths**	11,961	24,545	48,088	155,807
**QALYs gained**	56,056	191,318	551,824	2,465,008
**Healthcare cost-savings (dollars, billions)**^**c**^	5.28	10.56	19.69	41.93
**Policy administrative costs (dollars, millions)**	149	185	230	316
**Food subsidy costs (dollars, billions)**				
SNAP adults (age 35+ years)	−1.36	−2.54	−4.40	−6.04
All SNAP participants	−4.93	−8.96	−15.04	−21.51
**ICER (dollars/QALY), by perspective**^**d**^				
Societal	Saving ($5.23 billion)	Saving ($10.50 billion)	Saving ($19.61 billion)	Saving ($41.82 billion)
Government affordability (subsidizing SNAP adults aged 35+ years)	Saving ($6.60 billion)	Saving ($13.04 billion)	Saving ($24.01 billion)	Saving ($47.86 billion)
Government affordability (subsidizing all SNAP participants)	Saving ($10.16 billion)	Saving ($19.45 billion)	Saving ($34.65 billion)	Saving ($63.33 billion)

^a^Outcomes were evaluated among SNAP participants aged 35–80 years at baseline, corresponding to 14.5 million adults in 2017, followed until death or age 100, whichever occurred first. Compared to a base case of the current policy, the 3 interventions were (1) a 30% financial incentive for purchases of F&V (F&V incentive), (2) a 30% F&V incentive plus restriction of SSBs (F&V incentive/SSB restriction), and (3) a broader 30% incentive/disincentive program for multiple foods that preserves choice (SNAP-plus) including a 30% financial incentive for purchases of F&V, nuts, whole grains, fish, and plant-based oils and a 30% disincentive for purchases of SSBs, junk food, and processed meats (see S1 Table for food category details and definitions).

^b^Because we did not identify probable or convincing evidence of etiologic effects of F&V on type 2 diabetes [1] (see S3 Table), the F&V incentive in the model resulted in a slightly higher number of cases due to increased overall survival from prevented CVD.

^c^All costs were inflated to constant 2017 US dollars using the Bureau of Labor Statistics’ Consumer Price Index [42]. Costs and QALYs were discounted by 3% annually. Healthcare cost-savings were calculated as averted direct costs from chronic/acute disease states, surgical procedures, screening, and drug use. Food subsidy costs were evaluated for adult SNAP participants only (*N* = 14.6 million in 2017) and for all SNAP participants including children and adults aged <35 years (*N* = 42.1 million in 2017) [7,33].

^d^ICER thresholds were evaluated at $150,000/QALY and $50,000/QALY from 3 perspectives including (1) societal, (2) government affordability including subsidy costs for SNAP adults aged 35+ years, and (3) government affordability including subsidy costs for all SNAP participants including children and adults aged <35 years. As appropriate, the societal perspective did not include food subsidy costs or disincentive gains because these represent a transfer (like a tax or tax break) from one segment of society to another. Additional potential health benefits and healthcare cost-savings from these dietary interventions were conservatively excluded, including potential benefits for cancer in adults as well as all potential health benefits in children and young adults aged <35 years.

^e^Assuming that with full restriction, SNAP participants on average shift 50% of their SSB purchases in retail venues from SNAP dollars to other food dollars.

CVD, cardiovascular disease; F&V, fruits and vegetables; ICER, incremental cost-effectiveness ratio; NHANES, National Health and Nutrition Examination Survey; QALY, quality-adjusted life year; SNAP, Supplemental Nutrition Assistance Program; SSB, sugar-sweetened beverage; UI, uncertainty interval.

### Cost-effectiveness

From a societal perspective, considering both program implementation costs and healthcare savings, all 3 interventions were cost-saving at 5 years and beyond, with estimated lifetime societal savings of $6.69 billion, $39.05 billion, and $41.82 billion, respectively (Table 3; S1 Fig). From a government affordability perspective, incorporating the government costs of providing food subsidies, the F&V incentive was of low cost-effectiveness at 5 years, with ICERs of $204,928/QALY for subsidizing SNAP adults and $548,053/QALY for subsidizing all SNAP participants. However, by 10 years, the F&V incentive achieved traditional cost-effectiveness (<$150,000/QALY) for subsidizing SNAP adults, and over a lifetime, was very cost-effective (<$50,000/QALY) for subsidizing SNAP adults and cost-effective (<$150,000/QALY) for subsidizing all SNAP participants.

Adding SSB restriction produced a modest increase in policy administrative costs and (due to improved survival) increased F&V subsidy costs, but also a 4- to 5-fold increase in healthcare cost-savings (Table 3; S1 Fig). Consequently, from a government affordability perspective, the intervention was very cost-effective at 5 years and cost-saving at 10+ years for subsidizing SNAP adults (lifetime savings: $17.17 billion), and cost-effective at 10 years and very cost-effective at 20+ years, even when subsidizing all SNAP participants (lifetime ICER: $5,216/QALY).

For the SNAP-plus intervention, even with several more categories of incentivized foods, the food disincentives funded the incentives, creating a net program food cost-savings, or revenue. From a government affordability perspective, this resulted in a cost-saving intervention at all time points for subsidizing SNAP adults (lifetime savings: $27.74 billion) and for subsidizing all SNAP participants (lifetime savings: $63.33 billion). Evaluating costs within SNAP alone (i.e., ignoring healthcare savings), the SNAP-plus intervention remained cost-saving. For example, at 5 years, $0.15 billion in administrative costs was offset by 4.93 billion in savings from the combined food incentives/disincentives.

### Stratified and sensitivity analyses

**Fig 2:**
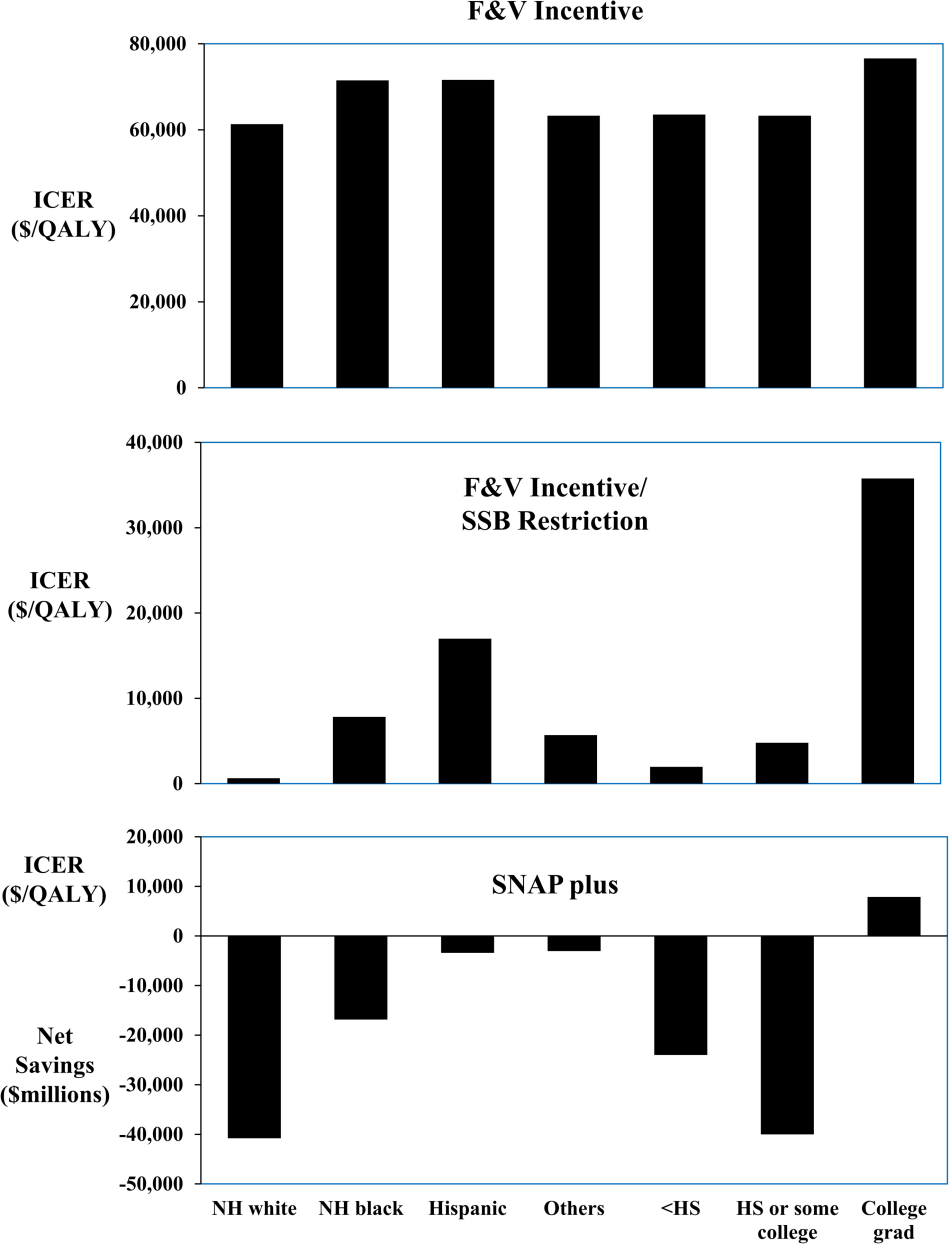
Lifetime cost-effectiveness of SNAP F&V incentive, F&V incentive and SSB restriction, and a combined incentive/disincentive program for multiple foods (SNAP-plus), by race/ethnicity and education. ICERs were calculated as the net change in costs divided by the net change in QALYs, compared to a base scenario of the current policy. Values are shown from a government affordability perspective, considering intervention and food subsidy costs for all SNAP participants including children and adults aged <35 years; SNAP-plus was net cost-saving in most subgroups. All scenarios were cost-saving from a societal perspective (not shown; see text).F&V, fruits and vegetables; HS, high school; ICER, incremental cost-effectiveness ratio; NH, non-Hispanic; QALY, quality-adjusted life year; SNAP, Supplemental Nutrition Assistance Program; SSB, sugar-sweetened beverage.

**Fig 3:**
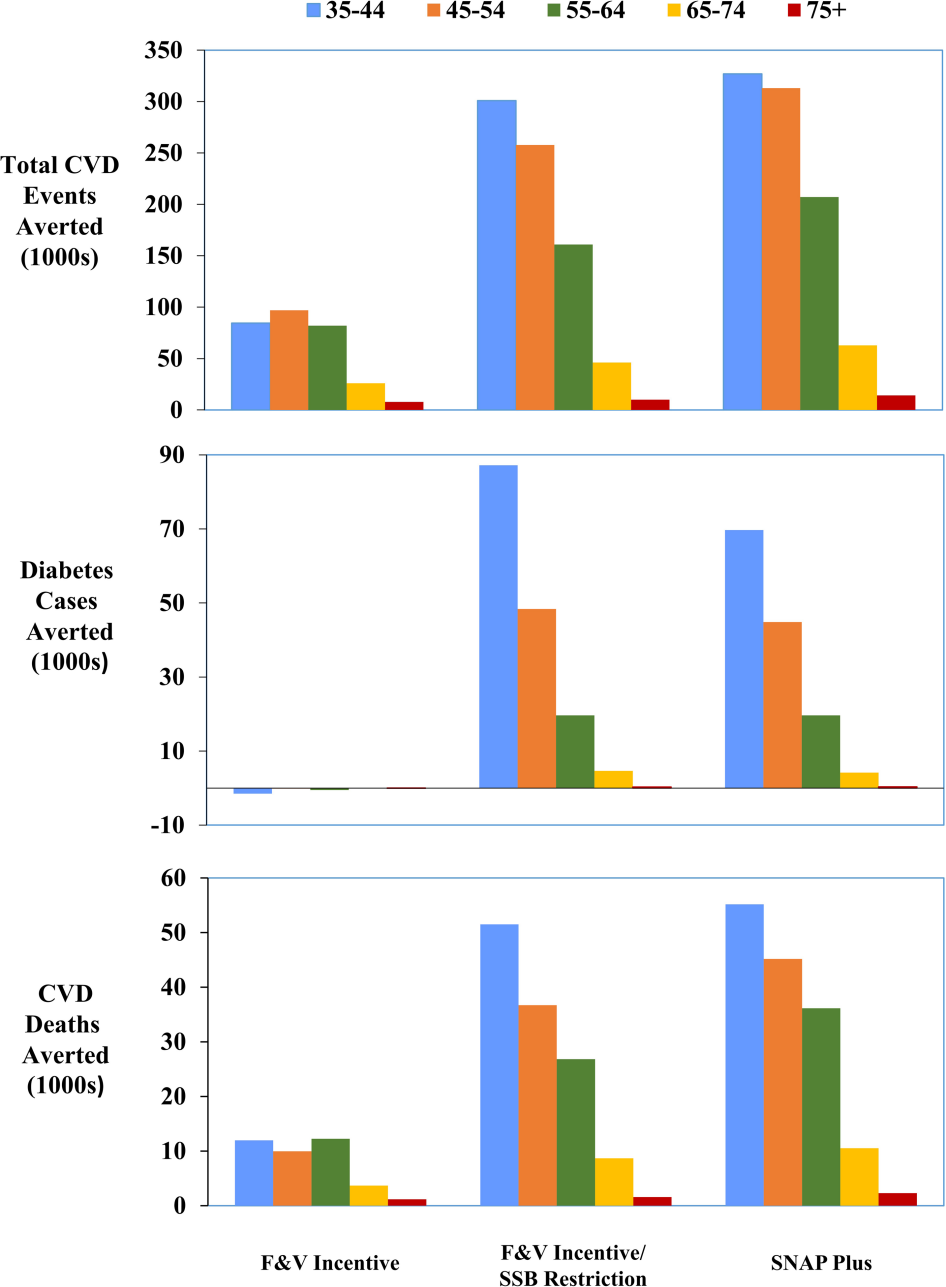
Lifetime averted total CVD events, diabetes cases, and CVD deaths by age (35–44, 45–54, 55–64, 65–74, 75+) across 3 intervention scenarios (i.e., F&V incentive, F&V incentive/SSB restriction, and SNAP-plus). CVD, cardiovascular disease; F&V, fruits and vegetables; SSB, sugar-sweetened beverage.

**Fig 4:**
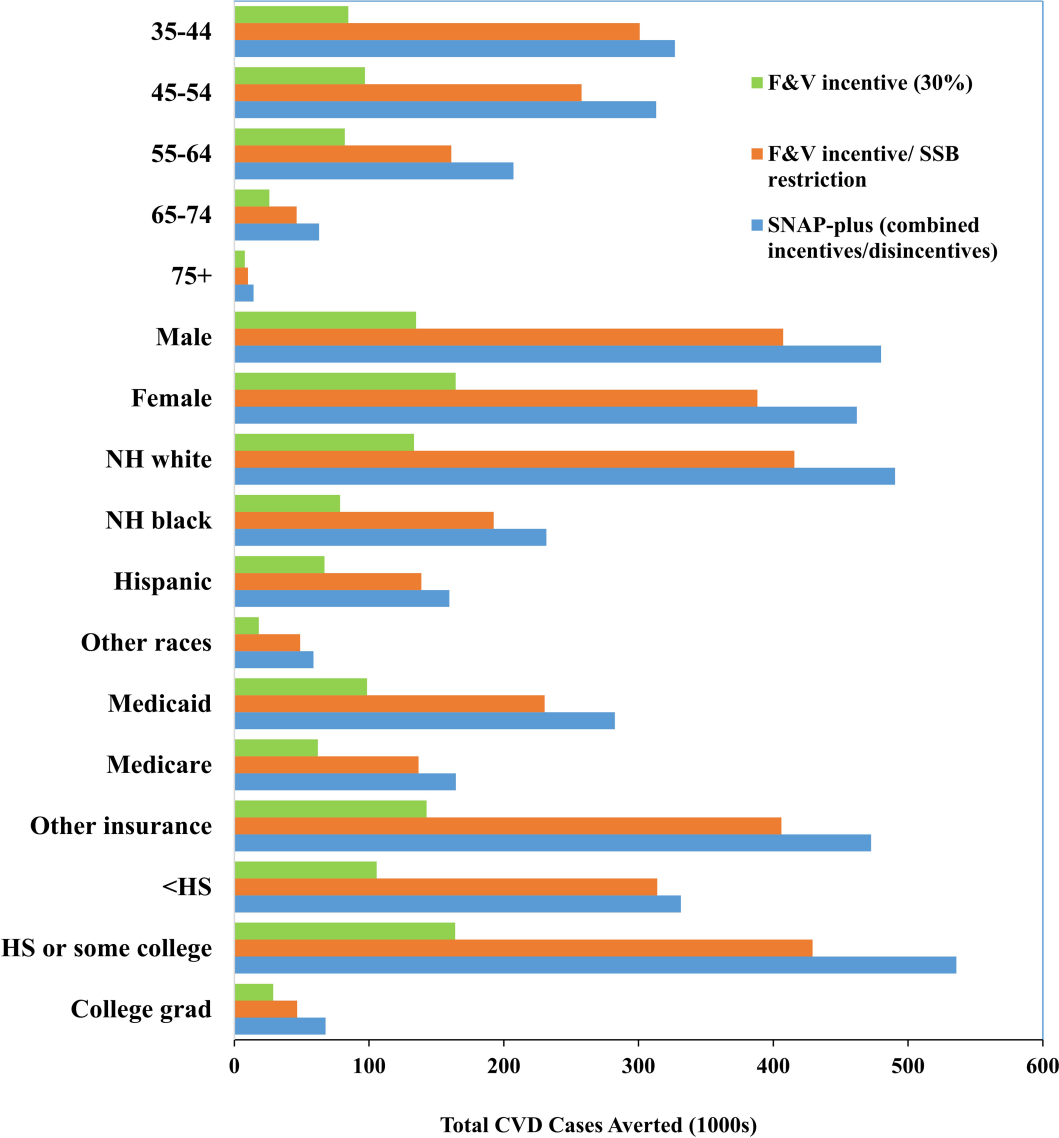
Lifetime averted total CVD cases by age (35–44, 45–54, 55–64, 65–74, 75+), sex (male, female), race/ethnicity (non-Hispanic white, non-Hispanic black, Hispanic, other), insurance status (Medicaid, Medicare, other), and education level (less than high school graduate, high school graduate or some college, college graduate) across 3 intervention scenarios (i.e., F&V incentive, F&V incentive/SSB restriction, and SNAP-plus). CVD, cardiovascular disease; F&V, fruits and vegetables; HS, high school; NH, non-Hispanic; SSB, sugar-sweetened beverage.

The cost-effectiveness or cost-savings and relative health gains of each intervention were generally similar across groups stratified by age, sex, race/ethnicity, education, and insurance status (Figs 2–4; S8 Table). For example, lifetime ICERs for the F&V incentive ranged from a low of $50,867/QALY in men to a high of $78,688/QALY in women from a government affordability perspective (subsidizing all SNAP participants). Adding SSB restriction substantially lowered ICERs in all groups, and produced net cost-savings in young adults (age 35–44 y) and in men. SNAP-plus was cost-saving in essentially all subgroups and for all perspectives, except for among college graduates from a government affordability perspective (subsidizing all SNAP participants), in whom the lifetime ICER was still very cost-effective, at $7,878/QALY.

**Fig 5:**
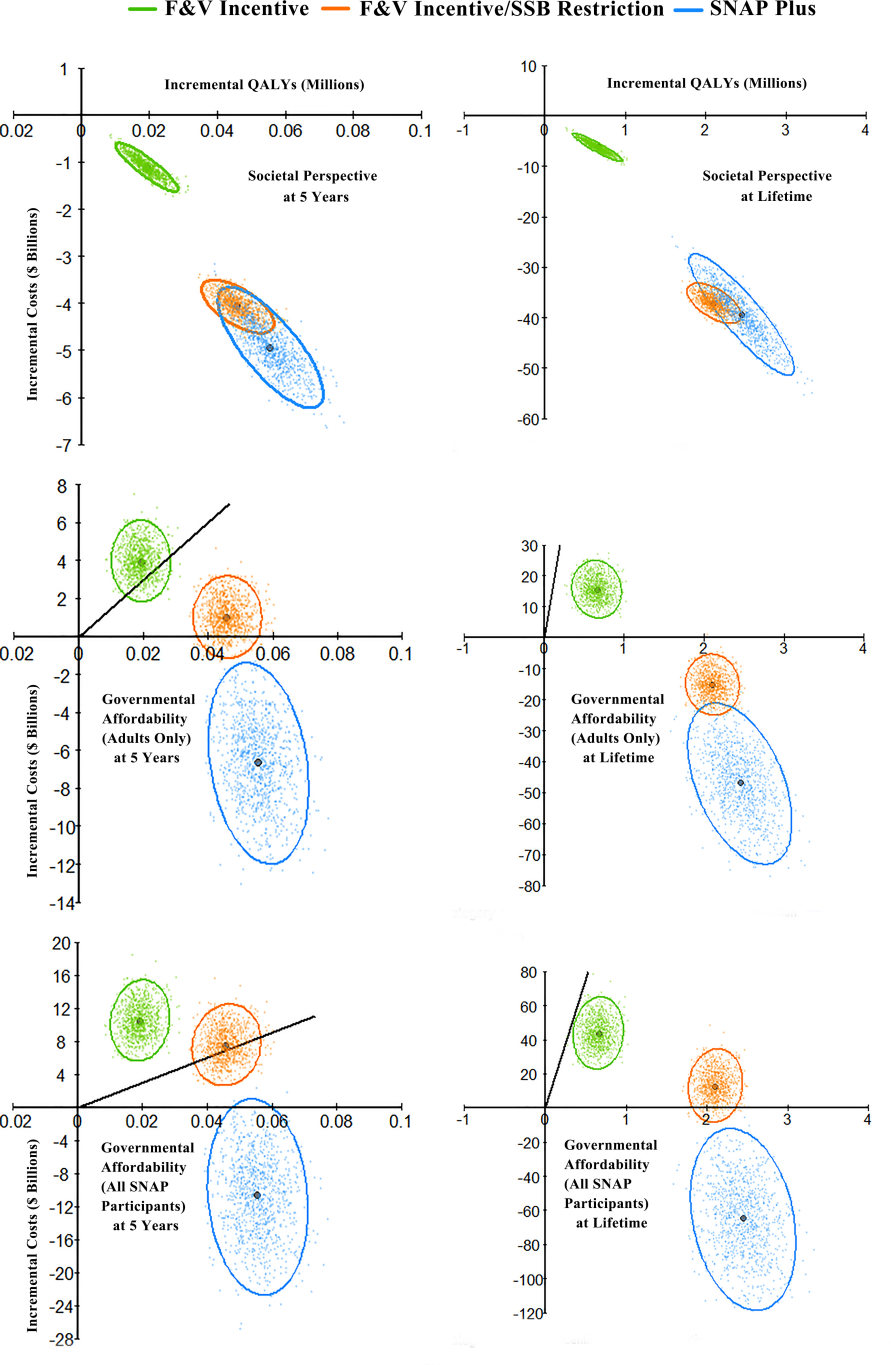
Probabilistic sensitivity analyses for cost-effectiveness of the SNAP F&V incentive, F&V incentive and SSB restriction, and a combined incentive/disincentive program for multiple foods (SNAP-plus), at 5 years and over a lifetime. Values are presented in cost-effectiveness planes of incremental costs ($ billions) versus incremental quality-adjusted life years (QALYs), compared to a base scenario of usual care. For each scenario, each colored dot depicts 1 of 1,000 Monte Carlo iterations, with the large dot depicting the median incremental cost-effectiveness ratio (ICER, $/QALY); and the ellipse depicting the 95% UIs. Results are presented from the perspective of society (top panels), government affordability including food subsidy costs for SNAP adults only (middle panels), and government affordability including food subsidy costs for all SNAP participants (bottom panels). Negative costs represent cost savings. The diagonal solid black lines represent a value of $150,000/QALY, a recommended threshold for assessing health interventions, with values to the right of the line being cost-effective with an ICER<$150,000/QALY. Note: due to different minimum and maximum quantities among panel figures, different scales were applied to each figure. F&V, fruits and vegetables; HS, high school; ICER, incremental cost-effectiveness ratio; NH, non-Hispanic; QALY, quality-adjusted life year; SNAP, Supplemental Nutrition Assistance Program; SSB, sugar-sweetened beverage.

In probabilistic sensitivity analyses, SNAP-plus was cost-saving both at 5 years and over a lifetime in 1,000 of 1,000 simulations (100%) from both societal and government affordability perspectives (Fig 5; S10 Table). The F&V incentive and F&V incentive/SSB restriction policies were also each cost-saving in 100% of simulations both at 5 years and over a lifetime from the societal perspective. From a government affordability perspective incorporating food subsidy costs for all SNAP participants including children and adults aged <35 years, the F&V incentive did not achieve cost-effectiveness (<$150,000/QALY) in any simulation at 5 years, but achieved cost-effectiveness in 99.9% of simulations over a lifetime, while the F&V incentive/SSB restriction policy was cost-saving or cost-effective in 44.6% of simulations at 5 years, and 100% over a lifetime.

Additional sensitivity analyses are shown in S7 Table. For the F&V incentive/SSB restriction policy, altering the assumed proportion of SSB purchases that would shift from SNAP dollars to other food dollars did not greatly alter the inference on this intervention. Similarly, for SNAP-plus, assuming a larger shift in purchases of disincentivized foods from SNAP dollars to other food dollars retained cost-savings at all time points for all perspectives. Removing subsidies in SNAP-plus for fish and plant-based oils, the most costly items, resulted in even larger cost-savings, with $80.77 billion in lifetime savings from a government affordability perspective (subsidizing all SNAP participants).

## Discussion

Based on our microsimulation model and nationally representative data, a F&V incentive, F&V incentive with SSB restriction, and combined incentive/disincentive program for multiple foods within SNAP would each generate significant health gains among low-income Americans. Our simulation estimated that the F&V subsidy would gain about 650,000 QALYs, while the other interventions would gain about 2.1 and 2.5 million QALYs. All interventions were cost-saving from a societal perspective, with estimated lifetime societal savings of about $6.7 billion, $39 billion, and $42 billion, respectively. From a government affordability perspective, all 3 programs met traditional cost-effectiveness thresholds over a lifetime, with superior cost-effectiveness when SSB restrictions were added to the F&V incentive, and net government cost-savings with SNAP-plus. Notably, the most conservative government affordability perspective incorporated the intervention and food subsidy costs for all SNAP participants, including children and young adults, while estimating health and healthcare cost benefits only for adults aged 35 years and older. Because consuming healthier diets at younger ages is likely to lead to health benefits, the overall health gains and cost-effectiveness or cost-savings of each intervention are likely to be underestimated.

The current average SNAP benefit is $126 per person per month, or about $4.20 per day [7]. Disparities in diet by SNAP status persist [12]. In 2015, the USDA launched the SNAP Food Insecurity Nutrition Incentive grant program, providing up to $31.5 million over 2 years for point-of-purchase incentives to increase F&V purchases [43]. Although this represents an important new commitment to leveraging SNAP for healthier choices and better well-being, our analysis suggests this amount—less than 50 cents per SNAP participant per year—is a small fraction of the approximately $2.6 billion/year needed to provide a 30% F&V incentive to all SNAP participants. In the current era of budget constraints, achieving political consensus for such an increase may be challenging. Incorporation of the associated health gains and healthcare cost-savings, as in our analysis, provides new evidence to inform this discussion. From a societal perspective, such an intervention was estimated to prevent tens of thousands of CVD events, gain substantial QALYs, and be cost-saving. From a government affordability perspective, a universal 30% F&V incentive in SNAP became cost-effective long term, although the shorter-term costs per QALY may not be attractive for resource-constrained decisions on short-term policy priorities.

Our findings suggest further health gains, cost-savings, and cost-effectiveness of adding SSB restriction to the F&V incentive in SNAP. While several groups have recommended SSB restrictions in SNAP [18,20,22,23], others have opposed such a change. In 2015, the bipartisan National Commission on Hunger recommended removing SSBs from the list of eligible purchases in SNAP [23]. Both the technology and precedent exist for altering product eligibility (e.g., alcohol and pre-prepared hot foods cannot be purchased in SNAP), and several states including California, Florida, Missouri, Wisconsin, and Texas have considered pilot programs to test restriction of SSBs [44]. However, such programs have never yet been federally authorized. SSB manufacturers oppose such a restriction [45], which appears to unfairly single out one product as compared to candy or other junk food, for example. Some anti-poverty advocates and ethical scholars argue that such a restriction is paternalistic, creating “a public policy message that poor people require government intervention to manage their food choices whereas higher-income persons do not” [44]. Our findings provide new data in this regard, estimating that adding SSB restriction to the F&V incentive would prevent an additional 494,000 CVD events and gain 1.46 million more QALYs among low-income Americans on SNAP. Government affordability also improved to about $5,000/QALY, superior to many other federally approved health interventions including drug treatment of hypertension ($20,000/QALY [46]), use of statins for primary prevention ($37,000/QALY [28]), and adding PCSK9 inhibitors to statins in patients with CVD ($414,000/QALY) or heterozygous familial hypercholesterolemia ($503,000/QALY) [47].

Our results suggest that a combined food incentive/disincentive program may be most attractive. The F&V incentive has widespread appeal yet was costly, while SSB restriction produced additional important health gains and cost-savings but raises political challenges in isolating a single product category and restricting choice. In comparison, the SNAP-plus program incentivized a broad range of foods, with partial disincentives but not absolute restrictions on less healthy products, preserving participant choice. The bipartisan National Commission on Hunger recommended incentivizing F&V, whole grains, high-quality proteins, and other healthful foods in SNAP [23]. Our results indicate that adding disincentives greatly augments both the health gains and affordability of such an intervention. Overall, the SNAP-plus intervention produced the largest gains in health and healthcare savings, and was cost-saving at all time points from all perspectives. Omitting healthcare savings and considering SNAP fiscal integrity alone—e.g., such as might be done by the Congressional Budget Office—the SNAP-plus intervention resulted in net savings of $4.78 billion at 5 years and $21.19 billion over a lifetime, funds that could be reinvested in SNAP to maintain or expand overall benefits. Novel online and technology-based financial incentive and education programs for healthier food purchases are now available for higher-income Americans through worksite wellness, health insurance, and life insurance programs [32]. Such technologies could be leveraged to provide SNAP participants with education and combined financial incentives and disincentives for healthier eating, with evaluation in state pilot programs.

Our investigation has several strengths. We utilized a validated microsimulation model and national data, increasing confidence in the validity and generalizability of our estimates. We accounted for proportions of food purchased from SNAP-eligible venues (as opposed to restaurants, cafeterias, etc.), proportions of purchases using SNAP versus other food dollars, and potential shifts in spending between SNAP and other food dollars, each reducing the overall effect and providing more appropriate and conservative estimates of potential impact. The potential effects of dietary substitutes and complements were also considered because the diet–disease etiologic effects (relative risks) for changes in the targeted dietary factors were based on long-term prospective cohort studies, which implicitly incorporate additional health effects of the average dietary substitutes and complements across the population. Policy intervention effects were based on trials among SNAP participants or, when these not available, prospective interventions and studies in general populations. We assessed both shorter- and longer-term health impacts, costs, and cost-effectiveness, providing a range of results across different potential time periods of interest.

Potential limitations should be considered. Our model cannot prove the health and cost impacts of national SNAP interventions. Rather, the estimates provide evidence that can be considered and incorporated into the design of state waiver programs for evaluation. Although prior interventions observed little shifting of spending dollars to maximize incentives [33], we cannot exclude that, over many years, participants might shift more of their spending on disincentivized foods to other food dollars, reducing intervention efficacy and cost-effectiveness over time. Conversely, establishing incentives and disincentives on specific food categories could also shift long-term knowledge and social norms around food purchases, increasing the efficacy and cost-effectiveness of the intervention over time. While our estimated etiologic effects for dietary changes inherently incorporated the average dietary complements and substitutes in the population, health gains could be augmented by additional intervention components aiming to encourage or discourage specific complements and substitutes. Our CVD risk calculations incorporated differences between people in age, sex, systolic blood pressure, total cholesterol, HDL-cholesterol, smoking status, diabetes status, baseline diet, and dietary changes due to the intervention, but not race/ethnicity. However, the risk factors in our model capture many of the major determinants (or further correlate with many of the determinants) that explain racial/ethnic disparities in disease risk, especially when further restricted by income (SNAP eligibility) as in our analysis. Our comparative effectiveness framework also minimizes the influence of potential residual heterogeneity in CVD risk, as uncaptured factors would influence both each intervention scenario and the base case scenario, having relatively small effects on the incremental comparison. We did not incorporate potential additional benefits of synchronizing use of SNAP-Ed, an existing $400 million/year educational program, with these financial incentive/disincentive programs. We conservatively excluded indirect costs of disease states, such as estimated losses in productivity, which, if included, would make each of the interventions more cost-effective or cost-saving.

In conclusion, our modeling estimates that leveraging SNAP for improved nutrition through financial incentives for healthier foods, and disincentives or altered eligibility for unhealthy foods, would generate significant health gains and healthcare cost-savings.

## Supporting information

S1 Fig Summary of health and cost-effectiveness findings for 3 dietary policy interventions in SNAP. A 30% F&V incentive, a 30% F&V incentive with SSB restriction, and a combined 30% incentive/disincentive program for multiple foods that preserves choice (SNAP-plus). Findings are shown at 5 years, 10 years, and 20 years and over a lifetime.
(TIF)

S1 Table Food categories for financial incentives or disincentives in SNAP. (DOCX)

S2 Table Sources and calculations for intervention effect sizes of incentives and disincentives applied in each of the scenarios in SNAP. (DOCX)

S3 Table Estimated etiologic effects of dietary components on cardiometabolic outcomes, by age. (DOCX)

S4 Table Multivariable associations of junk food consumption with other dietary factors linked to cardiometabolic risk, based on dietary data among US adults aged 35+ years in NHANES 2009–2014. (DOCX)

S5 Table Estimated intervention costs to implement financial incentive and disincentive policies through SNAP. (DOCX)

S6 Table Estimated healthcare costs. (DOCX)

S7 Table Sensitivity analyses of estimated cost-effectiveness of SNAP food subsidies, restrictions, and incentives/disincentives over 5, 10, and 20 years and over a lifetime. (DOCX)

S8 Table Lifetime estimated health gains, costs, and cost-effectiveness of each SNAP intervention, by age, sex, race, education, and insurance. (DOCX)

S9 Table Model inputs for probabilistic sensitivity analyses. (DOCX)

S10 Table Probabilistic sensitivity analyses at 5 years and over a lifetime. (DOCX)

S11 Table Comparison of relative risks for CHD observed in prospective cohort studies of dietary patterns and estimated relative risks for individual dietary factors. (DOCX)

S12 Table Comparison of relative risks for CHD calculated based on changes in systolic blood pressure and LDL-cholesterol in randomized controlled feeding trials of dietary patterns versus estimated relative risks for individual dietary factors. (DOCX)

S13 Table Comparison of relative risks for CHD observed in a large randomized clinical trial of dietary patterns versus estimated relative risks for individual dietary factors. (DOCX)

S1 Text CVD-PREDICT microsimulation model. (DOCX)

S2 Text Changes over time in SNAP participation. (DOCX)

S3 Text Shifts in food purchases between SNAP versus non-SNAP dollars. (DOCX)

S4 Text Assessment of validity and bias in diet–disease etiologic effects. (DOCX)

## Abbreviations

BMI: body mass index
CHD: coronary heart disease
CVD: cardiovascular disease
EBT: electronic benefits transfer
F&V: fruits and vegetables
HDL-cholesterol: high-density lipoprotein cholesterol
HIP: Healthy Incentives Pilot
ICER: incremental cost-effectiveness ratio
LDL-cholesterol: low-density lipoprotein cholesterol
NHANES: National Health and Nutrition Examination Survey
QALY: quality-adjusted life year
SNAP: Supplemental Nutrition Assistance Program
SSB: sugar-sweetened beverage
UCP: Universal Product Code
UI: uncertainty interval
USDA: US Department of Agriculture
