# Supplementary figures and images for: Cost-effectiveness of financial incentives and disincentives for improving food purchases and health through the US Supplemental Nutrition Assistance Program (SNAP): A microsimulation study
Source: PLoS Med. 2018 Oct 2;15(10):e1002661. doi: 10.1371/journal.pmed.1002661 (PMC6168180; doi:10.1371/journal.pmed.1002661)

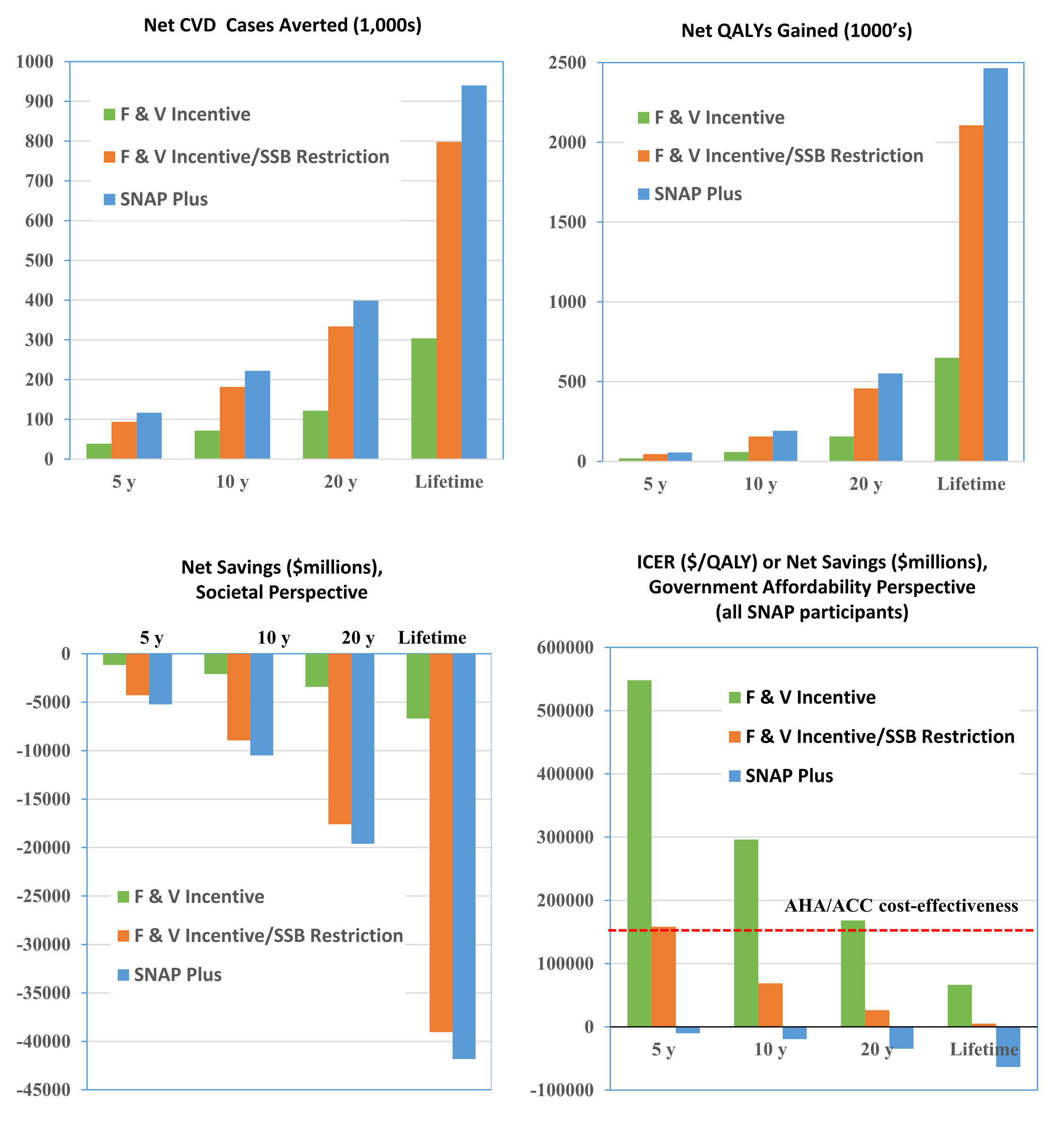

Supplement: S1 Fig — A 30% F&V incentive, a 30% F&V incentive with SSB restriction, and a combined 30% incentive/disincentive program for multiple foods that preserves choice (SNAP-plus). Findings are shown at 5 years, 10 years, and 20 years and over a lifetime. (TIF) [file pmed.1002661.s001.tif]
