# Supplementary material for: Cost-effectiveness of financial incentives and disincentives for improving food purchases and health through the US Supplemental Nutrition Assistance Program (SNAP): A microsimulation study
Source: PLoS Med. 2018 Oct 2;15(10):e1002661. doi: 10.1371/journal.pmed.1002661 (PMC6168180; doi:10.1371/journal.pmed.1002661)
Supplement: S1 Table — (DOCX) [file pmed.1002661.s002.docx]

# **S1 Table.** Food Categories for Financial Incentives or Disincentives in the Supplemental Nutrition Assistance Program (SNAP).*

| **Food Category** | **Included Foods** | **Excluded Foods** |
| --- | --- | --- |
| **Incentives** |  |  |
| **Fruits** | - Fresh, frozen, cooked, canned, dried fruits | - Fruit flavored drinks - Salted or pickled fruit - Fruit juice |
|  |  |  |
| **Vegetables** | - Fresh, frozen, cooked, canned, dried vegetables - Beans, legumes - Tomato sauce - Starchy vegetables except white potatoes | - Ketchup, barbeque, steak sauce - Salted or pickled vegetables (i.e., olives, pickles) - White potatoes - Vegetable juice |
|  |  |  |
| **Whole grains** | - Products containing the entire grain kernel (bran, germ, endosperm), such as amaranth, barley (not pearled), brown rice, buckwheat, bulgur, millets, oats, quinoa, dark rye, triticale, whole-grain wheat flour, whole-grain cracked wheat, wild rice, and grain-based products made with 100% whole grains or their flours | - Corn products (i.e. corn flour, corn meal, and popcorn) |
|  |  |  |
| **Nuts** | - Nuts, peanuts, and seeds, including nut, peanut, and seed butters | - Coconut |
|  |  |  |
| **Fish** | - Fish and shellfish | - Battered fish, fish sticks |
|  |  |  |
| **Plant-based oils** | - All plant based oils including vegetable, almond, coconut, corn, canola, peanut, olive, rapeseed, soybean, sesame, safflower, walnut, cottonseed, flaxseed, sunflower, and wheat germ oils. Health effects were based on polyunsaturated fat (PUFA) content of each. | - Palm oil |
| **Disincentives** |  |  |
| **Sugar-sweetened**  **beverages**† | - Soft, carbonated, sports, energy, and vitamin water drinks - Fruit drinks/nectars/squashes, lemonade, frescas, sugar cane beverages - Presweetened iced tea and coffee - Punch and other non-alcoholic drinks with added sugar (e.g. alcohol-free wines, alcohol-free malt beverages) | - 100% fruit & vegetable juice - Alcoholic beverages - Milk, milk-based drinks - Plant-based milk substitutes - Dietary supplements, beverages for medical use - Oral electrolyte solutions - Infant formula |
|  |  |  |
| **Junk food** ¶ | - Candies and sweets - Cakes, cookies, pies, pastries and bars - Crackers and salted snacks from grain products - Milk desserts and puddings - Potato chips | Potentially healthier options in these categories ¶ |
|  |  |  |
| **Processed meats** | - Bacon, salami, sausages, hot dogs, processed deli or luncheon meats | - Smoked or salted fish or eggs |

*Derived using data from NHANES 2009-2014, based on two 24-hour dietary recalls per person.

†Containing at least 5 g of added sugar per 12 oz, consistent with certain local and state SSB tax bills that have been proposed or implemented in the US.[1]

**¶**Junk food was defined according to the specified categories. To allow for potentially healthy options in these categories, we excluded any products with higher quality fats (for products with 5% or greater energy from fat), defined as an unsaturated to saturated fatty acid ratio of 4:1 or higher; higher quality carbohydrates (for products with 10% of greater energy from carbohydrate), defined as a carbohydrate to fiber ratio of 10:1 or lower; and with sodium content at or below the 2-year category-specific draft FDA voluntary sodium guidelines.[[2](#_ENREF_24)]

**References**

1. City of Boulder Colorado. Sugar Sweetened Beverage Tax2018 Jan 24, 2018. Available from: <https://bouldercolorado.gov/tax-license/finance-sugar-sweetened-beverage-tax>.

2. US Food and Drug Administration. Draft Guidance for Industry: Voluntary Sodium Reduction Goals: Target Mean and Upper Bound Concentrations for Sodium in Commercially Processed, Packaged, and Prepared Foods2016 Jan 24, 2018. Available from: <https://www.fda.gov/Food/GuidanceRegulation/GuidanceDocumentsRegulatoryInformation/ucm494732.htm>.
