# Supplementary material for: Cost-effectiveness of financial incentives and disincentives for improving food purchases and health through the US Supplemental Nutrition Assistance Program (SNAP): A microsimulation study
Source: PLoS Med. 2018 Oct 2;15(10):e1002661. doi: 10.1371/journal.pmed.1002661 (PMC6168180; doi:10.1371/journal.pmed.1002661)
Supplement: S2 Table — (DOCX) [file pmed.1002661.s003.docx]

# **S2 Table.** Sources and Calculations for Intervention Effect Sizes of Incentives and Disincentives Applied in Each of the Scenarios in Supplemental Nutrition Assistance Program (SNAP).

|  | Change in intake for a 30% price change ^a^ | Percent purchased using SNAP dollars ^b^ | Percent purchased at SNAP venues ^c^ | 1- % shift from SNAP to other food dollars ^d^ | Overall effect on diet ^e^ |
| --- | --- | --- | --- | --- | --- |
|  | | | | | |
| **30% incentive for fruits and vegetables** | | | | | |
| **Base-case** | | | | | |
| Fruits, servings/d | 26% (HIP[1]) | Incorporated in HIP estimate | 90% | Incorporated in HIP estimate | **+23.4%** |
| Vegetables, servings/d | 26% (HIP[1]) | Incorporated in HIP estimate | 73% | Incorporated in HIP estimate | **+19.0%** |
|  |  |  |  |  |  |
| **Alternative assumptions (for comparative purposes only)** | | | | | |
| Fruits, servings/d | 43% (meta-analysis) | 80% | 90% | 1 - 0% ^f^ | **+31.0%** |
| Vegetables, servings/d | 43% (meta-analysis) | 80% | 73% | 1 - 0% ^f^ | **+25.1%** |
|  |  |  |  |  |  |
| **Restriction of sugar-sweetened beverages (SSBs)** | | | | | |
| **Base-case** | | | | | |
| SSBs, servings/d | 100% (restriction) | 80% | 83% | 1 - 50% ^g^ | **-33.2%** |
| **Alternative assumptions (used in sensitivity analysis)** | | | | | |
| 25% shifting | 100% (restriction) | 80% | 83% | 1 - 25% | **-49.8%** |
| 75% shifting | 100% (restriction) | 80% | 83% | 1 - 75% | **-16.6%** |
|  |  |  |  |  |  |
| **30% incentive for whole grains, nuts/seeds, seafood, and plant-based oils** | | | | | |
| **Base-case** | | | | | |
| Fruits, servings/d | 26% (HIP) | Incorporated in HIP estimate | 90% | Incorporated in HIP estimate | **+23.4%** |
| Vegetables, servings/d | 26% (HIP) | Incorporated in HIP estimate | 73% | Incorporated in HIP estimate | **+19.0%** |
| Whole grains, serving/d | 26% (HIP) | Incorporated in HIP estimate | 93% | Incorporated in HIP estimate | **+24.2%** |
| Nuts, servings/d | 26% (HIP) | Incorporated in HIP estimate | 92% | Incorporated in HIP estimate | **+23.9%** |
| Fish, servings/d | 26% (HIP) | Incorporated in HIP estimate | 73% | Incorporated in HIP estimate | **+19.0%** |
| Plant-based oils, servings/d | 26% (HIP) | Incorporated in HIP estimate | 78% | Incorporated in HIP estimate | **+20.3%** |
|  |  |  |  |  |  |
| **30% disincentive for SSBs, junk food, and processed meats** | | | | | |
| **Base-case** | | | | | |
| SSBs, servings/d | 20% (meta-analysis) | 80% | 83% | 1 - 0% ^h^ | **-13.3%** |
| Processed meats, servings/d | 26% (meta-analysis) | 80% | 80% | 1 - 0% | **-16.9%** |
| Junk food, grams/d | 26% (meta-analysis) | 80% | 82% | 1 - 0% | **-17.4%** |
| **Alternative assumptions (used in sensitivity analysis)** | | | | | |
| SSB | 20% (meta-analysis) | 80% | 83% | 1 - 25% | **-10.0%** |
| Processed meats | 26% (meta-analysis) | 80% | 80% | 1 - 25% | **-12.5%** |
| Junk food | 26% (meta-analysis) | 80% | 82% | 1 - 25% | **-13.0%** |

^a^ Based on the Healthy Incentives Pilot (HIP) intervention trial[[1](#_ENREF_25),[2](#_ENREF_26)] or meta-analysis of interventional and prospective observational studies.[3]

^b^ Based on empirical evidence among SNAP participants that, on average, 80% of total food spending comes from SNAP dollars and 20% from other food dollars.[4] In the HIP trial, the effect of the 30% SNAP program subsidy was assessed for total purchases of fruits and vegetables from supermarkets and other venues accepting EBT cards, using all dollars. Thus, this effect size from HIP already incorporates the average percent of spending using SNAP dollars vs. other food dollars at these venues.

^c^ Based on empirical evidence from U.S. adult SNAP participants in NHANES 2009-14. The percentage of each food category consumed from supermarkets, grocery stores, convenience stores, and others stores (i.e., venues where the EBT card would be used) was calculated as a proportion of the total consumed (i.e., as compared to other locations such as restaurants, worksites, food pantries, etc.), utilizing NHANES survey and sampling weights.

^d^ Based on economic theory, SNAP incentives or disincentives may cause individuals to shift their spending between SNAP vs. other food dollars. For example, with a SNAP incentive, individuals may choose to maximize benefit by shifting purchases of incentivized item from other food dollars to SNAP dollars, which would increase the policy effect. The reverse might occur with a SNAP disincentive, which would reduce the policy effect. However, a specific analysis of shifting in the HIP trial demonstrated very little evidence of actual shifting due to incentives. In addition, because the main effect measure in HIP was the effect of the SNAP program subsidy on total purchases of fruits and vegetables from supermarkets and other venues accepting EBT cards, using all dollars, the effect size already incorporates the average shifting of spending at these venues using SNAP vs. other dollars.

^e^ For each food item, the multiplicative sum of the change in intake for a 30% price change, the percent of purchased food using SNAP dollars, the percent of purchased food at SNAP venues, and 1 minus the percent shifting of spending from SNAP dollars to other food dollars.

^f^ If any shifting in response to the intervention, the overall effect on diet would be even larger.

^g^ Although the HIP trial empirically demonstrated little shifting with incentives, we assumed that complete SSB restriction would lead to some shifting of SSB purchases from SNAP dollars to other food dollars. We used a central (base-case) assumption of 50% shifting (i.e., 50% of prior SNAP purchases of SSBs would shift to other food dollars), and sensitivity analyses of 25% and 75% shifting.

^h^ Because the HIP trial empirically demonstrated little shifting of purchases between SNAP dollars and other food dollars with a 30% incentive, our central (base-case) assumption was of no appreciable shifting with a 30% disincentive. In sensitivity analyses, we evaluated a 25% shift in spending.

**References**

1. Bartlett S, Klerman J, Olsho L, Logan C, Blocklin M, Beauregard M, et al. Evaluation of the Healthy Incentives Pilot (HIP): Final Report Alexandria, VA: USDA Food and Nutrition Service; 2014 [cited 2015 September 30]. Available from: <http://www.fns.usda.gov/sites/default/files/HIP-Final.pdf>.

2. Olsho LE, Klerman JA, Wilde PE, Bartlett S, Harnack L, Oakes JM, et al. Financial incentives increase fruit and vegetable intake among Supplemental Nutrition Assistance Program participants: a randomized controlled trial of the USDA Healthy Incentives Pilot. Am J Clin Nutr. 2016;104(2):423-35. Epub 2015/04/08

2016/06/24

2016/09/23. doi: 10.1016/j.ypmed.2015.03.019

10.3945/ajcn.115.129320

10.1001/jamainternmed.2016.5633. PubMed PMID: 27334234; PubMed Central PMCID: PMC4466151.

3. Afshin A, Penalvo JL, Del Gobbo L, Silva J, Michaelson M, O'Flaherty M, et al. The prospective impact of food pricing on improving dietary consumption: A systematic review and meta-analysis. PLoS One. 2017;12(3):e0172277. Epub 2017/03/02. doi: 10.1371/journal.pone.0172277. PubMed PMID: 28249003; PubMed Central PMCID: PMCPMC5332034.

4. Beatty TK, Tuttle CJ. Expenditure response to increases in in-kind transfers: Evidence from the Supplemental Nutrition Assistance Program. American Journal of Agricultural Economics. 2014;97(2):390-404.
