# Supplementary material for: Cost-effectiveness of financial incentives and disincentives for improving food purchases and health through the US Supplemental Nutrition Assistance Program (SNAP): A microsimulation study
Source: PLoS Med. 2018 Oct 2;15(10):e1002661. doi: 10.1371/journal.pmed.1002661 (PMC6168180; doi:10.1371/journal.pmed.1002661)
Supplement: S3 Table — (DOCX) [file pmed.1002661.s004.docx]

# **S3 Table.** Estimated Etiologic Effects of Dietary Components on Cardiometabolic Outcomes, by Age. ^a^

| **Dietary Component** | **Cardiometabolic outcome** | **Unit of Effect** | **Estimated Etiologic Effect (95% CI), by Age** | | | | | |
| --- | --- | --- | --- | --- | --- | --- | --- | --- |
|  |  |  | **25-34y** | **35-44y** | **45-54y** | **55-64y** | **65-74y** | **75+y** |
| Fruits | ↓ CHD | RR per 100 g/d | 0.92 | 0.92 | 0.93 | 0.94 | 0.95 | 0.97 |
|  |  |  | (0.87, 0.97) | (0.87, 0.97) | (0.89, 0.97) | (0.91, 0.98) | (0.92, 0.98) | (0.96, 0.99) |
|  | ↓ ischemic stroke |  | 0.83 | 0.83 | 0.86 | 0.88 | 0.9 | 0.94 |
|  |  |  | (0.76, 0.90) | (0.77, 0.90) | (0.80, 0.92) | (0.83, 0.93) | (0.86, 0.94) | (0.92, 0.96) |
|  | ↓ hemorrhagic stroke |  | 0.63 | 0.64 | 0.69 | 0.73 | 0.77 | 0.86 |
|  |  |  | (0.49, 0.81) | (0.5, 0.82) | (0.56, 0.84) | (0.61, 0.87) | (0.67, 0.89) | (0.8, 0.92) |
| Vegetables | ↓ CHD | RR per 100 g/d | 0.93 | 0.93 | 0.94 | 0.95 | 0.96 | 0.98 |
|  |  |  | (0.89, 0.97) | (0.9, 0.97) | (0.91, 0.97) | (0.93, 0.98) | (0.94, 0.98) | (0.97, 0.99) |
|  | ↓ ischemic stroke |  | 0.76 | 0.77 | 0.8 | 0.83 | 0.86 | 0.92 |
|  |  |  | (0.64, 0.9) | (0.66, 0.9) | (0.7, 0.92) | (0.74, 0.93) | (0.78, 0.94) | (0.87, 0.96) |
|  | ↓ hemorrhagic stroke |  | 0.76 | 0.77 | 0.8 | 0.83 | 0.86 | 0.92 |
|  |  |  | (0.61, 0.95) | (0.62, 0.95) | (0.67, 0.96) | (0.72, 0.96) | (0.76, 0.97) | (0.86, 0.97) |
| Nuts | ↓ CHD | RR per 1-oz serving/wk | 0.89 | 0.89 | 0.91 | 0.92 | 0.93 | 0.96 |
|  |  |  | (0.85, 0.93) | (0.85, 0.93) | (0.87, 0.94) | (0.89, 0.95) | (0.91, 0.96) | (0.95, 0.97) |
|  | ↓ diabetes |  | 0.95 | 0.95 | 0.96 | 0.97 | 0.97 | 0.98 |
|  |  |  | (0.92, 0.98) | (0.93, 0.98) | (0.94, 0.98) | (0.95, 0.98) | (0.96, 0.99) | (0.98, 0.99) |
| Whole grains | ↓ CHD | RR per 50 g/d | 0.95 | 0.95 | 0.96 | 0.97 | 0.97 | 0.98 |
|  |  |  | (0.91, 0.99) | (0.92, 0.99) | (0.93, 0.99) | (0.94, 0.99) | (0.95, 0.99) | (0.97, 0.99) |
|  | ↓ total stroke |  | 0.88 | 0.88 | 0.9 | 0.91 | 0.93 | 0.96 |
|  |  |  | (0.80, 0.96) | (0.81, 0.96) | (0.83, 0.97) | (0.86, 0.97) | (0.88, 0.98) | (0.93, 0.98) |
|  | ↓ diabetes |  | 0.83 | 0.83 | 0.86 | 0.88 | 0.90 | 0.94 |
|  |  |  | (0.76, 0.90) | (0.77, 0.90) | (0.80, 0.92) | (0.83, 0.93) | (0.83, 0.94) | (0.92, 0.96) |
| Red meats | ↑ diabetes | RR per 100 g/d | 1.3 | 1.29 | 1.24 | 1.19 | 1.16 | 1.09 |
|  |  |  | (1.05, 1.60) | (1.05, 1.57) | (1.04, 1.47) | (1.03, 1.37) | (1.03, 1.30) | (1.02, 1.15) |
| Processed meats | ↑ CHD | RR per 50 g/d | 1.62 | 1.58 | 1.47 | 1.38 | 1.30 | 1.16 |
|  |  |  | (1.17, 2.18) | (1.16, 2.11) | (1.14, 1.88) | (1.11, 1.69) | (1.09, 1.54) | (1.07, 1.27) |
|  | ↑ diabetes |  | 1.86 | 1.81 | 1.65 | 1.52 | 1.41 | 1.22 |
|  |  |  | (1.38, 2.46) | (1.36, 2.37) | (1.30, 2.08) | (1.24, 1.83) | (1.20, 1.65) | (1.12, 1.32) |
| PUFA replacing carbs | ↓ CHD | RR per 5%E | 0.86 | 0.86 | 0.88 | 0.9 | 0.92 | 0.95 |
|  |  |  | (0.79, 0.92) | (0.8, 0.93) | (0.83, 0.94) | (0.86, 0.95) | (0.88, 0.96) | (0.93, 0.97) |
| Seafood omega-3 fats | ↓ CHD (fatal) ^b^ | RR per 100 mg/d | 0.79 | 0.80 | 0.82 | 0.85 | 0.87 | 0.93 |
|  |  |  | (0.70, 0.88) | (0.71, 0.89) | (0.75, 0.90) | (0.79, 0.92) | (0.82, 0.93) | (0.90, 0.96) |
| Sodium | ↑ SBP, main effect, white, normotensive | mm Hg per 2300 mg/d | 1.64 | 2.69 | 3.74 | 4.79 | 5.84 | 5.84 |
|  |  |  | (-0.19, 3.46) | (1.15, 4.23) | (2.30, 5.17) | (3.25, 6.33) | (4.01, 7.66) | (4.01, 7.66) |
|  | ↑ SBP, additional effect among Blacks |  | 2.49 | 2.49 | 2.49 | 2.49 | 2.49 | 2.49 |
|  |  |  | (0.13, 4.85) | (0.13, 4.85) | (0.13, 4.85) | (0.13, 4.85) | (0.13, 4.85) | (0.13, 4.85) |
|  | ↑ SBP, additional effect among hypertensives |  | 1.87 | 1.87 | 1.87 | 1.87 | 1.87 | 1.87 |
|  |  |  | (0.12, 3.63) | (0.12, 3.63) | (0.12, 3.63) | (0.12, 3.63) | (0.12, 3.63) | (0.12, 3.63) |
| Potassium | ↓ total stroke | RR per 1,000 mg/d | 0.70  (0.57, 0.86) | 0.71  (0.58, 0.87) | 0.75  (0.63, 0.89) | 0.79  (0.68, 0.90) | 0.82  (0.73, 0.92) | 0.89  (0.84, 0.94) |
| SSBs | ↑ CHD, BMI-adjusted | RR per 8-oz serving/d | 1.33 | 1.31 | 1.26 | 1.21 | 1.17 | 1.09 |
|  |  |  | (1.19, 1.47) | (1.18, 1.45) | (1.15, 1.37) | (1.13, 1.3) | (1.10, 1.24) | (1.06, 1.13) |
|  | ↑ diabetes, BMI-adjusted |  | 1.35 | 1.33 | 1.27 | 1.22 | 1.18 | 1.10 |
|  |  |  | (1.14, 1.59) | (1.13, 1.56) | (1.11, 1.46) | (1.09, 1.36) | (1.07, 1.29) | (1.05, 1.15) |
|  | ↑ BMI (baseline BMI <25) | kg/m^2^ per 8-oz serving/d | 0.10 | 0.10 | 0.10 | 0.10 | 0.10 | 0.10 |
|  |  |  | (0.05, 0.15) | (0.05, 0.15) | (0.05, 0.15) | (0.05, 0.15) | (0.05, 0.15) | (0.05, 0.15) |
|  | ↑ BMI (baseline BMI ≥25) |  | 0.23 | 0.23 | 0.23 | 0.23 | 0.23 | 0.23 |
|  |  |  | (0.14, 0.32) | (0.14, 0.32) | (0.14, 0.32) | (0.14, 0.32) | (0.14, 0.32) | (0.14, 0.32) |
| Added sugar (non-SSB) ^c^ | ↑ BMI (baseline BMI <25) | kg/m^2^ per 10 g/d | 0.05 | 0.05 | 0.05 | 0.05 | 0.05 | 0.05 |
|  |  |  | (0.025, 0.075) | (0.025, 0.075) | (0.025, 0.075) | (0.025, 0.075) | (0.025, 0.075) | (0.025, 0.075) |
|  | ↑ BMI (baseline BMI ≥25) |  | 0.115 | 0.115 | 0.115 | 0.115 | 0.115 | 0.115 |
|  |  |  | (0.07, 0.16) | (0.07, 0.16) | (0.07, 0.16) | (0.07, 0.16) | (0.07, 0.16) | (0.07, 0.16) |
| BMI-mediated effects (for SSBs, added sugars) | ↑ diabetes | RR per 5 kg/m^2^ BMI increase | 3.55 | 3.07 | 2.66 | 2.32 | 2.03 | 1.52 |
|  |  |  | (2.41, 5.23) | (2.28, 4.15) | (2.15, 3.30) | (2.04, 2.63) | (1.95, 2.11) | (1.40, 1.65) |
|  | ↑ CHD, diabetes-adjusted |  | 1.45 | 1.42 | 1.35 | 1.28 | 1.23 | 1.13 |
|  |  |  | (1.36, 1.53) | (1.35, 1.51) | (1.29, 1.41) | (1.23, 1.33) | (1.19, 1.27) | (1.11, 1.14) |
|  | ↑ total stroke, diabetes-adjusted |  | 1.24 | 1.23 | 1.19 | 1.16 | 1.13 | 1.07 |
|  |  |  | (1.16, 1.33) | (1.15, 1.32) | (1.13, 1.26) | (1.10, 1.21) | (1.09, 1.17) | (1.05, 1.09) |

^a^ The detailed methods for reviewing and synthesizing evidence to estimate effect sizes for associations between dietary factors and cardiometabolic endpoints have been reported.[[1](#_ENREF_8),[2](#_ENREF_29)] We utilized evidence from meta-analyses of prospective coh**o**rts or randomized clinical trials evaluating direct associations of dietary factors with CHD, stroke, or type 2 diabetes, by age; associations of SSBs and added sugar from other foods with BMI and additional BMI-independent associations of SSBs with CHD and diabetes, by age and overweight/obesity status; and effects of dietary sodium on blood pressure, by age, race, and hypertensive status.

^b^ The available evidence suggests an effect of seafood omega-3 on fatal CHD, with less clear evidence for benefits on nonfatal CHD.[[3](#_ENREF_30)] Because the risk transitions influenced by diet in the CVD-Predict model are for incidence of a CHD event, with subsequent transitions to death independent of dietary risk factors (see Fig 1), the current analysis will modestly overestimate the benefits of changes in seafood omega-3 consumption.

^c^ For added sugar, we utilized data on BMI-mediated effects of SSBs,[[2](#_ENREF_29)] which were more conservative than effect sizes reported in a meta-analysis of added sugar and body weight.[[4](#_ENREF_31)] We assumed no additional effects of non-SSB added sugars on CHD, stroke, or diabetes beyond those mediated by changes in BMI.

BMI=body mass index. CHD=coronary heart disease. DM=type 2 diabetes. SBP=systolic blood pressure. SSB=sugar-sweetened beverage. RR=relative risk.

**References**

1. Micha R, Shulkin ML, Penalvo JL, Khatibzadeh S, Singh GM, Rao M, et al. Etiologic effects and optimal intakes of foods and nutrients for risk of cardiovascular diseases and diabetes: Systematic reviews and meta-analyses from the Nutrition and Chronic Diseases Expert Group (NutriCoDE). PLoS One. 2017;12(4):e0175149. Epub 2017/04/28. doi: 10.1371/journal.pone.0175149. PubMed PMID: 28448503; PubMed Central PMCID: PMCPMC5407851.

2. Micha R, Penalvo JL, Cudhea F, Imamura F, Rehm CD, Mozaffarian D. Association Between Dietary Factors and Mortality From Heart Disease, Stroke, and Type 2 Diabetes in the United States. JAMA. 2017;317(9):912-24. Epub 2017/03/08. doi: 10.1001/jama.2017.0947. PubMed PMID: 28267855; PubMed Central PMCID: PMCPMC5852674.

3. Mozaffarian D, Rimm EB. Fish intake, contaminants, and human health: evaluating the risks and the benefits. JAMA. 2006;296(15):1885-99. PubMed PMID: 17047219.

4. Te Morenga L, Mallard S, Mann J. Dietary sugars and body weight: systematic review and meta-analyses of randomised controlled trials and cohort studies. BMJ. 2013;346:e7492. Epub 2013/01/17. doi: 10.1136/bmj.e7492. PubMed PMID: 23321486.
