# Supplementary material for: Cost-effectiveness of financial incentives and disincentives for improving food purchases and health through the US Supplemental Nutrition Assistance Program (SNAP): A microsimulation study
Source: PLoS Med. 2018 Oct 2;15(10):e1002661. doi: 10.1371/journal.pmed.1002661 (PMC6168180; doi:10.1371/journal.pmed.1002661)
Supplement: S4 Table — (DOCX) [file pmed.1002661.s005.docx]

# **S4 Table.** Multivariable Associations of Junk Food Consumption with Other Dietary Factors Linked to Cardiometabolic Risk, based on Dietary Data among U.S. Adults Age 35+ Years in NHANES 2009-2014. ^a^

| **Dietary components linked to cardiometabolic risk** | **Difference in consumption of each dietary factor per each 100 g/d of junk food consumption** |
| --- | --- |
| Vegetables, g/d | -28 (-33, -23) |
| Nuts, g/d | -3.5 (-5.5, -1.5) |
| Seafood omega-3, mg/d | -27 (-34, -20) |
| Red meats, g/d | -5.0 (-7.0, -2.8) |
| Processed meats, g/d | -2.3 (-3.8, -0.90) |
| SSBs, 8-fl-oz serving/d | -0.05 (-0.11, -0.001) |
| Added sugar, g/d | +14 (+12, +16) |
| Sodium, mg/d | -220 (-283, -158) |
| Potassium, mg/d | -160 (-191, -129) |

^a^ Based on multivariable-adjusted linear regression including adjustment for age, sex, race, and 13 dietary components linked to cardiometabolic risk,[[1](#_ENREF_8)] including all components in the Table as well as fruits, whole grains, polyunsaturated fat replacing carbohydrates or saturated fat, and dietary fiber. Intakes of the latter four components were not independently associated with junk food intake (not shown). Analyses utilized robust variance estimation and used NHANES survey weights to account for the complex survey design and sampling. Because associations of added sugar and sugar-sweetened beverages (SSBs) were in opposing directions, they were jointly included in the estimation of indirect etiologic effects of junk food. Using the identified significant associations between junk food intake and these 9 dietary components and their joint associations with cardiometabolic risk, we modeled the impact of changes in junk food intake on cardiometabolic risk. Some of these associations would predict cardiometabolic benefit from lower junk food intake (e.g., the inverse association with vegetables, nuts/seeds, seafood omega-3’s, and potassium; and the positive association with added sugars), while some would predict harm (e.g., the inverse association with red meat, processed meat, SSBs, and sodium).

**References**

1. Micha R, Shulkin ML, Penalvo JL, Khatibzadeh S, Singh GM, Rao M, et al. Etiologic effects and optimal intakes of foods and nutrients for risk of cardiovascular diseases and diabetes: Systematic reviews and meta-analyses from the Nutrition and Chronic Diseases Expert Group (NutriCoDE). PLoS One. 2017;12(4):e0175149. Epub 2017/04/28. doi: 10.1371/journal.pone.0175149. PubMed PMID: 28448503; PubMed Central PMCID: PMCPMC5407851.
