# Supplementary material for: Cost-effectiveness of financial incentives and disincentives for improving food purchases and health through the US Supplemental Nutrition Assistance Program (SNAP): A microsimulation study
Source: PLoS Med. 2018 Oct 2;15(10):e1002661. doi: 10.1371/journal.pmed.1002661 (PMC6168180; doi:10.1371/journal.pmed.1002661)
Supplement: S5 Table — (DOCX) [file pmed.1002661.s006.docx]

# **S5 Table.** Estimated Intervention Costs to Implement Financial Incentives and Disincentives Policies through Supplemental Nutrition Assistance Program (SNAP).^a^

| **Cost category** | **Cost** | | **Source** |
| --- | --- | --- | --- |
| **Administrative costs** ^b^ | Year 1 | Years 2+ |  |
| Initial program implementation and participant education | $30,521,533 | n/a | Healthy Incentives Pilot (HIP) Final Report [1] |
| Implementation of updated EBT processing and retail infrastructure | $69,684,913 | n/a | HIP Final Report [1] |
| Subsequent program administration, participant education, monitoring and evaluation ^c^ | n/a | $5,638,225 | Estimation based on the cost of the HIP evaluation |
|  |  |  |  |
| **Subsidy cost/disincentive revenue on dietary target** ^d^ | | | |
| Fruit (subsidy) | $0.33680 per 100 g | | USDA Economic Research Service (ERS) Quarterly Food-at-Home Price Database [2] |
| Vegetables (subsidy) | $0.28901 per 100 g | |  |
| Nuts (subsidy) | $0.76322 per 100 g | |  |
| Plant-based oils (subsidy) | $0.75732 per 100 g | |  |
| Fish (subsidy) | $1.15186 per 100 g | |  |
| Whole grains (subsidy) | $0.64420 per 100 g | |  |
| SSBs (disincentive) | $0.062925 per fl-oz | | Powell et al. 2012 [[3](#_ENREF_33)] |
| Junk food (disincentive) | $0.94408 per 100 g | | ERS Quarterly Food-at-Home Price Database [[2](#_ENREF_32)] |
| Processed meat (disincentive) | $0.61435 per 100 g | | Consumer Expenditure Survey[[4](#_ENREF_34)]; USDA SNAP report[[5](#_ENREF_35)] |

^a^ Administrative costs were derived from the HIP report estimates for extending the project nationally.[1] The costs of food incentives and disincentives were calculated for each food category based on data from the USDA Economic Research Service (ERS) Quarterly Food-at-Home Price Database,[[2](#_ENREF_32)] the estimated average national price of SSBs,[[3](#_ENREF_33)] the Consumer Expenditure Survey,[[4](#_ENREF_34)] and the USDA SNAP report.[[5](#_ENREF_35)] All costs were inflated to constant 2017 dollars using the Bureau of Labor Statistics’ Consumer Price Index.[[6](#_ENREF_36)]

^b^ Detailed cost estimates in 2012 dollars were extracted from the HIP report Exhibit 9.3 (cost of HIP implementation) and Exhibit 9.7 (estimated cost of a nationwide expansion of HIP) and summed into two categories: initial program implementation and participant education, and implementation of updated EBT processing and retail infrastructure. We assumed these costs would occur only in year 1, as the HIP report concluded that a national HIP program would not increase the staffing and operation cost of the SNAP EBT systems for states, EBT processors, and retailers on an ongoing basis.

^c^ In addition to initial implementation costs, ongoing costs were included to oversee, monitor and evaluate the programs. To estimates these costs, we referenced the cost to design and evaluate HIP. Abt Associates, the firm contracted by USDA’s Food and Nutrition Service to implement HIP, received $10,573,000 over 4 years to design and evaluate the pilot (Parke Wilde, personal communication, Jan 10, 2018), for an annual cost of $2,643,250. Assuming that evaluation will only be one aspect of ongoing program management, we doubled this amount to reflect the full estimated program management cost for the F&V incentive program. To account for the administrative complexity in the F&V incentive/SSB restriction and SNAP-plus, we quadrupled this amount to reflect the full estimated program management cost for these two scenarios. Some of these efforts could also be separately covered or expanded by SNAP-Ed, a $400 million/year program that allows states to teach people using or eligible for SNAP about good nutrition and how to make their food dollars stretch further. This could further increase the efficacy and cost-effectiveness of each intervention.

^d^ Average retail costs of foods in each category were estimated using the ERS Quarterly Food-At-Home Price Database version 1 (prices of random-weight foods were not included in version 2). We used 2006 data, stratified by region, and calculated the weighted national average price (unit cost) of each dietary component, inflated to constant 2017 dollars. For SSBs, we referenced the average price from Powell et al., who used nationally-representative data from the Bridging the Gap Community Obesity Measures Project, weighted proportionally to package size and source of consumption in NHANES 2009-2010.[[3](#_ENREF_33)] For processed meats, which were not included in the ERS database, the average price was estimated using the recent USDA report on SNAP food purchases,[[5](#_ENREF_35)] based on the annual, aggregated expenditure of different types of processed meat (e.g., lunchmeat, bacon, hot dogs, etc.) as a proportion of total spending on total meat, poultry, fish, and eggs (excluding spending on plant-based protein foods such as nuts, peanut butter, beans, etc.). This proportion (20.31%) was applied to the daily per-person expenditure on total meat, poultry, fish and eggs for consumers in the lowest income quartile, a proxy for SNAP participants, of the Consumer Expenditure Survey[[4](#_ENREF_34)] to estimate the per-person daily expenditure on processed meat. This value was divided by the per-person daily consumption of processed meat among SNAP adult participants in NHANES 2009-14 to estimate the average unit cost of processed meat in $/100g.

**References**

1. Bartlett S, Klerman J, Olsho L, Logan C, Blocklin M, Beauregard M, et al. Evaluation of the Healthy Incentives Pilot (HIP): Final Report Alexandria, VA: USDA Food and Nutrition Service; 2014 [cited 2015 September 30]. Available from: <http://www.fns.usda.gov/sites/default/files/HIP-Final.pdf>.

2. U.S. Department of Agriculture, Economic Research Service. Quarterly Food-at-Home Price Database. Available from: <https://www.ers.usda.gov/data-products/quarterly-food-at-home-price-database/>.

3. Powell L, Isgor Z, Rimkus L, Chaloupka F. Sugar-sweetened beverage prices: estimates from a national sample of food outlets. Bridging the Gap Program, Health Policy Center, Institute for Health Research and Policy, University of Illinois at Chicago, Chicago, IL. 2014.

4. Bureau of Labor Statistics, U.S. Department of Labor. Consumer Expenditure Survey2016 Jan 24, 2018. Available from: <https://www.bls.gov/cex/>.

5. Garasky S, Mbwana K, Romualdo A, Tenaglio A, Roy M. Foods Typically Purchased by Supplemental Nutrition Assistance Program (SNAP) Households 2016. Available from: <https://fns-prod.azureedge.net/sites/default/files/ops/SNAPFoodsTypicallyPurchased.pdf>.

6. U.S. Department of Labor. The Bureau of Labor Statistics' Consumer Price Index 2017 [cited 2017 October 16]. Available from: <https://www.bls.gov/data/#prices>.
