# Supplementary material for: Cost-effectiveness of financial incentives and disincentives for improving food purchases and health through the US Supplemental Nutrition Assistance Program (SNAP): A microsimulation study
Source: PLoS Med. 2018 Oct 2;15(10):e1002661. doi: 10.1371/journal.pmed.1002661 (PMC6168180; doi:10.1371/journal.pmed.1002661)
Supplement: S6 Table — (DOCX) [file pmed.1002661.s007.docx]

# **S6 Table.** Estimated Healthcare Costs.^a^

|  | **Cost, $** | **Source** |
| --- | --- | --- |
| **CVD costs:** |  |  |
| **Chronic disease states, per year** |  |  |
| Chronic coronary heart disease | 3,362 | Lee 2010 [2] |
| Chronic stroke | 2,222 | Pignone 2006 [3] |
| **Acute disease states** |  |  |
| Acute cardiac arrest | 20,242 | O’Sullivan 2011 [[4](#_ENREF_39)] |
| Acute myocardial infarction | 58,254 |  |
| Acute angina | 30,607 |  |
| Acute stroke | 20,092 |  |
| **Procedures and repeat events** |  |  |
| Repeat myocardial infarction | 58,254 | O’Sullivan 2011 [[4](#_ENREF_39)] |
| Repeat stroke | 20,092 |  |
| Coronary artery bypass graft | 38,730 |  |
| Percutaneous coronary intervention | 36,493 |  |
| **Screening** |  |  |
| GP visit in stage 1 | 79 | Pletcher 2009 [[5](#_ENREF_40)] |
| Cholesterol lab test | 37 |  |
| No. of extra GP visits during stage 2 | 1 | assumption |
| No. of lab tests/year after treatment | 1 | Lazar 2011 [6] |
| No. of GP visits/year after treatment | 1 |  |
| **Medications, per year** |  |  |
| Statin | 280 | Redbook 2009 [7] |
| Anti-hypertensive | 213 | Nuckols 2011 [8] |
| Aspirin | 8 | Pignone 2006 [3] |
| ACE inhibitor | 54 | Shah 2011 [9], Redbook 2009 [7] |
| Beta blocker | 54 |  |
| **Statin-associated adverse events** | |  |
| Mild adverse event | 185 | Lee 2010 [2] |
| Major adverse event | 7,280 |  |
|  |  |  |
| **Type 2 diabetes costs:** ^b^ |  |  |
| **Institutional care, per year**  Hospital inpatient  Nursing/residential facility  Hospice  **Outpatient care, per year**  Physician office  Emergency department  Ambulance services  Hospital outpatient  Home health  Podiatry  **Medications and supplies, per year**  Insulin  Diabetic supplies  Other antidiabetic agents  Other prescription medications  Other equipment and supplies | 2495  485  1  501  219  7  166  147  7  202  76  399  1043  35 | ADA 2013[10], Zhuo 2013[11] |

^a^ All costs inflated to constant 2017 dollars using the Bureau of Labor Statistics’ Consumer Price Index.[1]

^b^ Type 2 diabetes costs were derived from analyses by the American Diabetes Association (ADA),[10] modified to exclude costs for complications due to CVD based on an estimated 53% of type 2 diabetes-related lifetime medical costs being due to treating diabetic complications, of which 57% were due to CVD.[11]

ACE=angiotensin-converting enzyme inhibitor. GP=general practitioner.

**References**

1. U.S. Department of Labor. The Bureau of Labor Statistics' Consumer Price Index 2017 [cited 2017 October 16]. Available from: <https://www.bls.gov/data/#prices>.

2. Lee KK, Cipriano LE, Owens DK, Go AS, Hlatky MA. Cost-effectiveness of using high-sensitivity C-reactive protein to identify intermediate- and low-cardiovascular-risk individuals for statin therapy. Circulation. 2010;122(15):1478-87. Epub 2010/09/30. doi: 10.1161/circulationaha.110.947960. PubMed PMID: 20876434.

3. Pignone M, Earnshaw S, Tice JA, Pletcher MJ. Aspirin, statins, or both drugs for the primary prevention of coronary heart disease events in men: a cost-utility analysis. Ann Intern Med. 2006;144(5):326-36. Epub 2006/03/08. PubMed PMID: 16520473.

4. O'Sullivan AK, Rubin J, Nyambose J, Kuznik A, Cohen DJ, Thompson D. Cost estimation of cardiovascular disease events in the US. PharmacoEconomics. 2011;29(8):693-704. Epub 2011/05/19. doi: 10.2165/11584620-000000000-00000. PubMed PMID: 21585226.

5. Pletcher MJ, Lazar L, Bibbins-Domingo K, Moran A, Rodondi N, Coxson P, et al. Comparing impact and cost-effectiveness of primary prevention strategies for lipid-lowering. Ann Intern Med. 2009;150(4):243-54. Epub 2009/02/18. PubMed PMID: 19221376.

6. Lazar LD, Pletcher MJ, Coxson PG, Bibbins-Domingo K, Goldman L. Cost-effectiveness of statin therapy for primary prevention in a low-cost statin era. Circulation. 2011;124(2):146-53. Epub 2011/06/29. doi: 10.1161/circulationaha.110.986349. PubMed PMID: 21709063.

7. Thomson Corporation. Red book: Pharmacy's Fundamental Reference. Montvale, NJ Thomson PDR; 2009.

8. Nuckols TK, Aledort JE, Adams J, Lai J, Go MH, Keesey J, et al. Cost implications of improving blood pressure management among U.S. adults. Health services research. 2011;46(4):1124-57. Epub 2011/02/11. doi: 10.1111/j.1475-6773.2010.01239.x. PubMed PMID: 21306365; PubMed Central PMCID: PMCPMC3165181.

9. Shah ND, Mason J, Kurt M, Denton BT, Schaefer AJ, Montori VM, et al. Comparative effectiveness of guidelines for the management of hyperlipidemia and hypertension for type 2 diabetes patients. PLoS One. 2011;6(1):e16170. Epub 2011/02/02. doi: 10.1371/journal.pone.0016170. PubMed PMID: 21283569; PubMed Central PMCID: PMCPMC3026790.

10. Economic costs of diabetes in the U.S. in 2012. Diabetes Care. 2013;36(4):1033-46. Epub 2013/03/08. doi: 10.2337/dc12-2625. PubMed PMID: 23468086; PubMed Central PMCID: PMCPMC3609540.

11. Zhuo X, Zhang P, Hoerger TJ. Lifetime direct medical costs of treating type 2 diabetes and diabetic complications. Am J Prev Med. 2013;45(3):253-61. Epub 2013/08/21. doi: 10.1016/j.amepre.2013.04.017. PubMed PMID: 23953350.
