# Supplementary material for: Cost-effectiveness of financial incentives and disincentives for improving food purchases and health through the US Supplemental Nutrition Assistance Program (SNAP): A microsimulation study
Source: PLoS Med. 2018 Oct 2;15(10):e1002661. doi: 10.1371/journal.pmed.1002661 (PMC6168180; doi:10.1371/journal.pmed.1002661)
Supplement: S7 Table — (DOCX) [file pmed.1002661.s008.docx]

# **S7 Table.** Sensitivity Analyses of Estimated Cost-Effectiveness of SNAP Food Subsidies, Restrictions, and Incentives/Disincentives over 5, 10, 20 Years and Lifetime. ^a^

|  | **5 years** | | **10 years** | | **20 years** | | **Lifetime** |
| --- | --- | --- | --- | --- | --- | --- | --- |
| **F&V incentive/ SSB restriction** | | | | | | | |
| *25% shifting in SSB purchases ^b^* |  | |  | |  | |  |
| ICER ($/QALY), by perspective |  | |  | |  | |  |
| Societal | Saving ($5.52B) | | Saving ($11.63B) | | Saving ($23.16B) | | Saving ($52.24B) |
| Government affordability  (subsidizing SNAP adults age 35+) | Saving ($0.47B) | | Saving ($3.03B) | | Saving ($10.15B) | | Saving ($30.33B) |
| Government affordability  (subsidizing all SNAP participants) | 106,072 | | 40,928 | | 11,228 | | Saving ($2.13B) |
| *75% shifting in SSB purchases ^b^* | | | | | | | |
| ICER ($/QALY), by perspective |  | |  | |  | |  |
| Societal | Saving ($2.80B) | | Saving ($5.75B) | | Saving ($11.09B) | | Saving ($23.97B) |
| Government affordability  (subsidizing SNAP adults age 35+) | 67,967 | | 25,805 | | 5,995 | | Saving ($2.12B) |
| Government affordability  (subsidizing all SNAP participants) | 264,488 | | 125,987 | | 58,538 | | 18,392 |
|  |  | |  | |  | |  |
| **SNAP-plus (combined incentives/disincentives)** | | | | | | | |
| *25% shifting in disincentivized foods* ^c^ | | | | | | | |
| ICER ($/QALY), by perspective |  |  | |  | |  | |
| Societal | Saving ($4.59B) | Saving ($9.19B) | | Saving ($16.99B) | | Saving ($35.92B) | |
| Government affordability  (subsidizing SNAP adults age 35+) | Saving ($3.39B) | Saving ($7.25B) | | Saving ($14.36B) | | Saving ($30.73B) | |
| Government affordability  (subsidizing all SNAP participants) | Saving ($3.29B) | Saving ($7.33B) | | Saving ($15.13B) | | Saving ($30.24B) | |
| *Excluding subsidies for plant oils and fish* |  |  | |  | |  | |
| ICER ($/QALY), by perspective |  |  | |  | |  | |
| Societal | Saving ($4.49B) | Saving ($9.16B) | | Saving ($17.36B) | | Saving ($37.27B) | |
| Government affordability  (subsidizing SNAP adults age 35+) | Saving ($8.56B) | Saving ($16.49B) | | Saving ($29.53B) | | Saving ($55.20B) | |
| Government affordability  (subsidizing all SNAP participants) | Saving ($14.41B) | Saving ($26.89B) | | Saving ($46.45B) | | Saving ($80.77B) | |

**^a^** See Table 3 for details of methods, interventions, and inputs. Incremental cost-effectiveness ratio (ICER) thresholds were evaluated at $150,000/QALY and $50,000/QALY from three perspectives including (1) societal, (2) governmental affordability including subsidy costs for SNAP adults age 35+ y, and (3) government affordability including subsidy costs for all SNAP participants including children and adults age <35 y. As appropriate, the societal perspective did not include food subsidy costs or disincentive gains because these represent a transfer (like a tax or tax break) from one segment of society to another. Additional potential health benefits and healthcare cost-savings from these dietary interventions were conservatively excluded, including potential benefits for cancer in adults as well as all potential health benefits in children and young adults age <35 y.

^b^ Assuming that with full restriction, either 25% or 75% of purchases of SSBs from retail venues shift from SNAP dollars to participants’ other food dollars, compared to the base case (Table 3) of 50% shifting.

^c^ Assuming that with a 30% disincentive, 25% of purchases of disincentivized foods from retail venues shift from SNAP dollars to participants’ other food dollars, compared to the base case (Table 3) of no shifting. In practice, such shifts are expected to be small, based on empirical findings on virtually no shifting of spending in the Healthy Incentive Pilot trial.[1]

**References**

1. Wilde P, Klerman JA, Olsho LEW, Bartlett S. Explaining the Impact of USDA’s Healthy Incentives Pilot on Different Spending Outcomes. Applied Economic Perspectives and Policy. 2015;0(1-18). doi: 10.1093/aepp/ppv028.
