# Supplementary material for: Cost-effectiveness of financial incentives and disincentives for improving food purchases and health through the US Supplemental Nutrition Assistance Program (SNAP): A microsimulation study
Source: PLoS Med. 2018 Oct 2;15(10):e1002661. doi: 10.1371/journal.pmed.1002661 (PMC6168180; doi:10.1371/journal.pmed.1002661)
Supplement: S8 Table — (DOCX) [file pmed.1002661.s009.docx]

# **S8 Table.** Lifetime Estimated Health Gains, Costs, and Cost-Effectiveness of Each SNAP Intervention, by Age, Sex, Race, Education and Insurance.^a^

|  | **Cases Averted** | | | **Incremental QALYs** | **Incremental Cost ($M), by Perspective** ^b^ | | | **ICER ($/QALY), by Perspective** ^b^ | | |
| --- | --- | --- | --- | --- | --- | --- | --- | --- | --- | --- |
|  | **Total CVD** | **Diabetes** ^c^ | **CVD Deaths** |  | **Societal** | **Government affordability**  **(adults)** | **Government affordability**  **(all)** | **Societal** | **Government affordability**  **(adults)** | **Government affordability**  **(all)** |
| **F&V incentive (30%)** | | | | | |  |  |  |  |  |
| 35-44 years | 84,564 | -1498 | 11,977 | 184,068 | -$1,569 | $5,397 | $14,216 | Saving | 29,321 | 77,230 |
| 45-54 years | 96,928 | -148 | 9,961 | 199,308 | -$2,094 | $4,262 | $12,385 | Saving | 21,386 | 62,142 |
| 55-64 years | 81,986 | -547 | 12,249 | 207,868 | -$1,703 | $4,508 | $12,759 | Saving | 21,688 | 61,379 |
| 65-74 years | 26,026 | -91 | 3,690 | 56,796 | -$511 | $1,230 | $3,448 | Saving | 21,651 | 60,704 |
| 75+ years | 7,795 | -51 | 1,161 | 13,377 | -$149 | $342 | $988 | Saving | 25,600 | 73,836 |
| Men | 134,948 | -346 | 15,796 | 325,377 | -$3,122 | $5,509 | $16,551 | Saving | 16,931 | 50,867 |
| Women | 164,241 | 759 | 23,742 | 341,543 | -$3,342 | $9,838 | $26,875 | Saving | 28,804 | 78,688 |
| NH white | 133,374 | -1413 | 16,420 | 311,393 | -$2,979 | $6,745 | $19,097 | Saving | 21,660 | 61,329 |
| NH black | 78,439 | -909 | 9,560 | 151,430 | -$1,516 | $3,795 | $10,824 | Saving | 25,062 | 71,478 |
| Hispanic | 66,974 | -468 | 9,477 | 150,526 | -$1,347 | $3,992 | $10,780 | Saving | 26,520 | 71,615 |
| Other races | 18,229 | -238 | 2,542 | 42,108 | -$381 | $928 | $2,665 | Saving | 22,045 | 63,300 |
| < HS | 105,628 | -258 | 13,613 | 237,053 | -$2,177 | $5,414 | $15,064 | Saving | 22,840 | 63,549 |
| HS or some college | 163,846 | -2162 | 21,573 | 357,897 | -$3,362 | $8,028 | $22,650 | Saving | 22,432 | 63,286 |
| College grad | 28,796 | -467 | 5,527 | 79,090 | -$612 | $2,237 | $6,058 | Saving | 28,286 | 76,600 |
| Medicaid^d^ | 98,498 | -266 | 10,828 | 184,265 | -$1,961 | $4,570 | $12,949 | Saving | 24,802 | 70,272 |
| Medicare^e^ | 61,994 | -355 | 7,433 | 113,172 | -$1,193 | $2,721 | $7,853 | Saving | 24,044 | 69,392 |
| Others^f^ | 142,659 | -2,192 | 20,198 | 324,957 | -$2,976 | $7,875 | $21,817 | Saving | 24,235 | 67,138 |
| **F&V incentive/ SSB restriction** | | | | | | | | | | |
| 35-44 years | 300,913 | 87,205 | 51,513 | 814,180 | -$16,266 | -$9,274 | -$426 | Saving | Saving | Saving |
| 45-54 years | 257,755 | 48360 | 36,699 | 657,311 | -$12,304 | -$5,925 | $2,228 | Saving | Saving | 3,389 |
| 55-64 years | 161,033 | 19,665 | 26,818 | 457,093 | -$6,542 | -$316 | $7,955 | Saving | Saving | 17,403 |
| 65-74 years | 46,255 | 4,676 | 8,675 | 127,791 | -$1,731 | $13 | $2,236 | Saving | 104 | 17,498 |
| 75+ years | 10,070 | 300 | 1,598 | 18,374 | -$263 | $228 | $874 | Saving | 12,416 | 47,567 |
| Men | 407,341 | 92,057 | 66,939 | 1,226,320 | -$21,197 | -$12,524 | -$1,432 | Saving | Saving | Saving |
| Women | 388,213 | 76,958 | 60,086 | 895,761 | -$17,434 | -$4,226 | $12,846 | Saving | Saving | 14,341 |
| NH white | 415,617 | 91,879 | 66,250 | 1,172,345 | -$21,413 | -$11,655 | $739 | Saving | Saving | 631 |
| NH black | 192,468 | 38,434 | 28,524 | 450,399 | -$8,853 | -$3,527 | $3,523 | Saving | Saving | 7,822 |
| Hispanic | 138,762 | 28,377 | 23,382 | 354,988 | -$6,123 | -$770 | $6,034 | Saving | Saving | 16,998 |
| Other races | 48,820 | 8,664 | 8,320 | 139,514 | -$2,262 | -$948 | $794 | Saving | Saving | 5,693 |
| < HS | 313,888 | 67,626 | 52,546 | 867,794 | -$15,587 | -$7,966 | $1,720 | Saving | Saving | 1,982 |
| HS or some college | 429,137 | 90,112 | 68,928 | 1,124,228 | -$20,698 | -$9,273 | $5,391 | Saving | Saving | 4,796 |
| College grad | 46,650 | 7,168 | 9,125 | 135,044 | -$1,852 | $1,002 | $4,830 | Saving | 7,419 | 35,767 |
| Medicaid^d^ | 230,344 | 44,172 | 3,614 | 528,311 | -$10,321 | -$3,772 | $4,629 | Saving | Saving | 8,763 |
| Medicare^e^ | 136,694 | 23,179 | 20,466 | 312,121 | -$5,877 | -$1,953 | $3,191 | Saving | Saving | 10,223 |
| Others^f^ | 406,049 | 89,874 | 66,749 | 1,126,143 | -$20,344 | -$9,456 | $4,531 | Saving | Saving | 4,023 |
| **SNAP-plus (combined incentives/disincentives)** | | | | | | | | | | |
| 35-44 years | 327,007 | 69,680 | 55,195 | 850,993 | -15,711 | -19,112 | -26,525 | Saving | Saving | Saving |
| 45-54 years | 313,071 | 44,851 | 45,188 | 800,381 | -13,897 | -16,817 | -22,934 | Saving | Saving | Saving |
| 55-64 years | 207,195 | 19,643 | 36,132 | 602,745 | -8,218 | -8,030 | -9,187 | Saving | Saving | Saving |
| 65-74 years | 62,843 | 4,195 | 10,532 | 160,818 | -2,304 | -2,008 | -2,220 | Saving | Saving | Saving |
| 75+ years | 14,225 | 329 | 2,310 | 26,587 | -402 | -196 | -146 | Saving | Saving | Saving |
| Men | 479,975 | 78,723 | 78,996 | 1,419,950 | -22,849 | -27,344 | -36,217 | Saving | Saving | Saving |
| Women | 462,009 | 68,079 | 73,226 | 1,065,742 | -18,977 | -20,511 | -27,128 | Saving | Saving | Saving |
| NH white | 490,336 | 78,898 | 78,063 | 1,362,435 | -23,044 | -29,172 | -40,788 | Saving | Saving | Saving |
| NH black | 231,649 | 34,960 | 35,020 | 537,082 | -9,781 | -11,917 | -16,837 | Saving | Saving | Saving |
| Hispanic | 159,555 | 24,321 | 27,612 | 404,797 | -6,497 | -4,535 | -3,404 | Saving | Saving | Saving |
| Other races | 58,785 | 6,437 | 9,925 | 158,908 | -2,420 | -2,507 | -3,041 | Saving | Saving | Saving |
| < HS | 331,344 | 50,638 | 54,806 | 880,409 | -14,650 | -17,566 | -24,007 | Saving | Saving | Saving |
| HS or some college | 535,852 | 83,534 | 85,291 | 1,399,279 | -23,996 | -28,900 | -39,999 | Saving | Saving | Saving |
| College grad | 67,691 | 9,727 | 14,202 | 204,248 | -2,745 | -740 | 1,609 | Saving | Saving | 7,878 |
| Medicaid^d^ | 282,466 | 36,857 | 40,448 | 627,101 | -$11,498 | -$11,928 | -$15,211 | Saving | Saving | Saving |
| Medicare^e^ | 164,357 | 18,010 | 23,290 | 360,390 | -$6,442 | -$7,479 | -$9,838 | Saving | Saving | Saving |
| Others^f^ | 472,539 | 78,130 | 79,895 | 1,318,036 | -$21,764 | -$25,638 | -$34,403 | Saving | Saving | Saving |

^a^ The distribution of the population in each subgroup was derived from the survey weighted percentages among adults SNAP participants in NHANES 2009-2014 (see Table 1).

^b^ Incremental net costs and ICERs were evaluated from three perspectives. The societal perspective included program administration costs and healthcare savings, but did not incorporate food subsidy costs or disincentive gains because these represent a transfer (like a tax or tax break) from one segment of society to another. The first governmental affordability perspective included subsidy costs for SNAP adult participants age 35+ y. The second governmental affordability perspective included subsidy costs for all SNAP participants, including children and adults age <35 y. Additional potential health benefits and healthcare cost-savings from these dietary interventions were conservatively excluded, for example including potential benefits for cancer in adults as well as all potential health benefits in children and adults age <35 y.

^c^ Because we did not identify probable or convincing evidence of etiologic effects of fruits and vegetables on type 2 diabetes[[1](#_ENREF_8)] (see S3 Table), the F&V incentive resulted in slightly higher number of cases due to increased overall survival from prevented CVD.

^d^ SNAP participants on Medicaid, including dual eligibles (also on Medicare).

^e^ SNAP participants on Medicare, including dual eligibles (also on Medicaid).

^f^ SNAP participants on neither Medicaid nor Medicare.

HS=high school. NH=non-Hispanic.

**References**

1. Micha R, Shulkin ML, Penalvo JL, Khatibzadeh S, Singh GM, Rao M, et al. Etiologic effects and optimal intakes of foods and nutrients for risk of cardiovascular diseases and diabetes: Systematic reviews and meta-analyses from the Nutrition and Chronic Diseases Expert Group (NutriCoDE). PLoS One. 2017;12(4):e0175149. Epub 2017/04/28. doi: 10.1371/journal.pone.0175149. PubMed PMID: 28448503; PubMed Central PMCID: PMCPMC5407851.
