# Supplementary material for: Cost-effectiveness of financial incentives and disincentives for improving food purchases and health through the US Supplemental Nutrition Assistance Program (SNAP): A microsimulation study
Source: PLoS Med. 2018 Oct 2;15(10):e1002661. doi: 10.1371/journal.pmed.1002661 (PMC6168180; doi:10.1371/journal.pmed.1002661)
Supplement: S9 Table — (DOCX) [file pmed.1002661.s010.docx]

# **S9 Table.** Model Inputs for Probabilistic Sensitivity Analyses**.^a^**

| **Inputs** | **Mean value (SD)** | **Distribution** | **Source** |
| --- | --- | --- | --- |
| **Costs** ^b^ |  |  |  |
| One time implementation cost | 30,521,533 (7,630,383) | Gamma | HIP Final Report[[2](#_ENREF_25)] |
| One time infrastructure cost | 69,684,913 (17,421,228) | Gamma | HIP Final Report[[2](#_ENREF_25)] |
| Annual program management costs | 5,638,225 (1,409,556) | Gamma | Estimation based on the cost of the HIP evaluation |
| Unit food costs |  |  |  |
| Fruits ($/100g) | 0.33680 (0.084) | Gamma | USDA Economic Research Service (ERS) Quarterly Food-at-home Price Database[5] |
| Vegetables ($/100g) | 0.28901 (0.072) | Gamma |  |
| Whole grains ($/100g) | 0.6442 (0.003) | Gamma |  |
| Nuts ($/100g) | 0.7632 (0.191) | Gamma |  |
| Fish ($/100g) | 1.15186 (0.288) | Gamma |  |
| Plant-based oils ($/100g) | 0.75732 (0.189) | Gamma |  |
| SSBs ($/8 oz) | 0.5034 (0.126) | Gamma | Powell et al. 2012[6] |
| Junk food ($/100g) | 0.94408 (0.236) | Gamma | ERS Quarterly Food-at-home Price Database[5] |
| Processed meat ($/100g) | 0.61435(0.154) | Gamma | Consumer Expenditure Survey[7]; USDA SNAP report[8] |
| **Change in intake for a 30% price change for base case ^c^** |  |  |  |
| Fruits | 23.4% (6.3) | Normal | HIP[3,12] |
| Vegetables | 19.0% (5.1) | Normal | HIP[3,12] |
| Whole grains | 24.2% (6.5) | Normal | HIP[3,12] |
| Nuts | 23.9% (6.4) | Normal | HIP[3,12] |
| Fish | 19.0% (5.1) | Normal | HIP[3,12] |
| Plant-based oils | 20.3% (5.5) | Normal | HIP[3,12] |
| SSBs | -13.3% (-3.7) | Normal | Afshin et al. 2017[4] |
| Processed meats | -16.9% (-2.7) | Normal | Afshin et al. 2017[4] |
| Junk food | -17.4% (-2.8) | Normal | Afshin et al. 2017[4] |
| **Diet-CVD etiologic effects (RRs)** | Table S3 | Log-normal | Micha et al. 2017[13]  Lu et al., 2014[14] |
| **Procedural adverse events** | | | |
| Patients with adverse events, mean % | | | |
| Major | 0.006 (0.0003) | Beta | Zhang et al. 2013[15] |
| Probability major adverse event is fatal, mean % | 0.09 (0.0045) | Beta | Alsheikh-Ali et al. 2005 [16] |
| **CVD healthcare costs** | | | |
| General practitioner screening visit | 79 (7.67) | Gamma | RBRVS[17] |
| Cholesterol laboratory test | 37 (5.83) | Gamma | RBRVS[17] |
| **Acute** | | | |
| Cardiac arrest | 20,242 (963.55) | Gamma | O’Sullivan et al. 2011[11] |
| Fatal myocardial infarction | 18,129 (862.95) | Gamma | O’Sullivan et al. 2011[11] |
| Nonfatal myocardial infarction | 65,334 (3,100) | Gamma | O’Sullivan et al. 2011[11] |
| Angina | 30,607 (1,456) | Gamma | O’Sullivan et al. 2011[11] |
| Fatal stroke | 11,183 (532.35) | Gamma | O’Sullivan et al. 2011[11] |
| Nonfatal stroke | 21,542 (1,025.45) | Gamma | O’Sullivan et al. 2011[11] |
| Coronary artery bypass grafting | 38,730 (1,843.6) | Gamma | O’Sullivan et al. 2011[11] |
| Percutaneous coronary  angioplasty | 36,493 (1737.1) | Gamma | O’Sullivan et al. 2011[11] |
| Post-first-year annual cost | | | |
| CHD | 3,362(533.67) | Gamma | Lee et al., 2010[9] |
| Stroke | 2,222 (352.5) | Gamma | Pignone et al. 2006[10] |
| **Informal healthcare costs** | | | |
| Travel costs per outpatient visit | 8.8 (0.42) | Gamma | Russel et al. 2008[18] |
| Wait-time costs per outpatient visit | 10.6 (0.504) | Gamma | Russel et al. 2008[18] |
| **Utility weights** |  |  |  |
| Disease free | 1 | Beta | Assumption |
| Cardiac arrest | 0.808 (0.0404) | Beta | Sullivan et al. 2006[[1](#_ENREF_2)] |
| Myocardial infarction | 0.778(0.0389) | Beta | Sullivan et al. 2006[[1](#_ENREF_2)] |
| Angina | 0.768 (0.0384) | Beta | Sullivan et al. 2006[[1](#_ENREF_2)] |
| Stroke | 0.768 (0.0384) | Beta | Sullivan et al. 2006[[1](#_ENREF_2)] |

Abbreviations: SSB, sugar-sweetened beverages; CHD, coronary heart disease; CVD, cardiovascular disease; RBRVS, resource-based relative value scale.

^a^ Because the CVD-PREDICT model reports the average pooled population and stratum effects for each microsimulation, potential variation related to individual-level uncertainty in health state transitions was not incorporated and would have little influence on the pooled average findings.

^b^ SDs for costs were defined as 25% of the central cost estimate.

^c^ Based on the Healthy Incentives Pilot (HIP) intervention trial or meta-analysis of intervention and prospective observational studies.

**References**

1. Sullivan PW, Ghushchyan V. Preference-based EQ-5D index scores for chronic conditions in the United States. Medical Decision Making. 2006;26(4):410-20.

2. Bartlett S, Klerman J, Olsho L, Logan C, Blocklin M, Beauregard M, et al. Evaluation of the Healthy Incentives Pilot (HIP): Final Report Alexandria, VA: USDA Food and Nutrition Service; 2014 [cited 2015 September 30]. Available from: <http://www.fns.usda.gov/sites/default/files/HIP-Final.pdf>.

3. Olsho LE, Klerman JA, Wilde PE, Bartlett S, Harnack L, Oakes JM, et al. Financial incentives increase fruit and vegetable intake among Supplemental Nutrition Assistance Program participants: a randomized controlled trial of the USDA Healthy Incentives Pilot. Am J Clin Nutr. 2016;104(2):423-35. Epub 2015/04/08

2016/06/24

2016/09/23. doi: 10.1016/j.ypmed.2015.03.019

10.3945/ajcn.115.129320

10.1001/jamainternmed.2016.5633. PubMed PMID: 27334234; PubMed Central PMCID: PMC4466151.

4. Afshin A, Penalvo JL, Del Gobbo L, Silva J, Michaelson M, O'Flaherty M, et al. The prospective impact of food pricing on improving dietary consumption: A systematic review and meta-analysis. PLoS One. 2017;12(3):e0172277. Epub 2017/03/02. doi: 10.1371/journal.pone.0172277. PubMed PMID: 28249003; PubMed Central PMCID: PMCPMC5332034.

5. U.S. Department of Agriculture, Economic Research Service. Quarterly Food-at-Home Price Database. Available from: <https://www.ers.usda.gov/data-products/quarterly-food-at-home-price-database/>.

6. Powell L, Isgor Z, Rimkus L, Chaloupka F. Sugar-sweetened beverage prices: estimates from a national sample of food outlets. Bridging the Gap Program, Health Policy Center, Institute for Health Research and Policy, University of Illinois at Chicago, Chicago, IL. 2014.

7. Bureau of Labor Statistics, U.S. Department of Labor. Consumer Expenditure Survey2016 Jan 24, 2018. Available from: <https://www.bls.gov/cex/>.

8. Garasky S, Mbwana K, Romualdo A, Tenaglio A, Roy M. Foods Typically Purchased by Supplemental Nutrition Assistance Program (SNAP) Households 2016. Available from: <https://fns-prod.azureedge.net/sites/default/files/ops/SNAPFoodsTypicallyPurchased.pdf>.

9. Lee KK, Cipriano LE, Owens DK, Go AS, Hlatky MA. Cost-effectiveness of using high-sensitivity C-reactive protein to identify intermediate- and low-cardiovascular-risk individuals for statin therapy. Circulation. 2010;122(15):1478-87. Epub 2010/09/30. doi: 10.1161/circulationaha.110.947960. PubMed PMID: 20876434.

10. Pignone M, Earnshaw S, Tice JA, Pletcher MJ. Aspirin, statins, or both drugs for the primary prevention of coronary heart disease events in men: a cost-utility analysis. Ann Intern Med. 2006;144(5):326-36. Epub 2006/03/08. PubMed PMID: 16520473.

11. O'Sullivan AK, Rubin J, Nyambose J, Kuznik A, Cohen DJ, Thompson D. Cost estimation of cardiovascular disease events in the US. PharmacoEconomics. 2011;29(8):693-704. Epub 2011/05/19. doi: 10.2165/11584620-000000000-00000. PubMed PMID: 21585226.

12. Bartlett S KJ, Olsho L, et al. E. vaulation of the Healthy Incentives Pilot (HIP): Final Report. 2014; .

13. Micha R, Peñalvo JL, Cudhea F, Imamura F, Rehm CD, Mozaffarian D. Association Between Dietary Factors and Mortality From Heart Disease, Stroke, and Type 2 Diabetes in the United States. Jama. 2017;317(9):912-24.

14. Lu Y, Hajifathalian K, Ezzati M, Woodward M, Rimm EB, Danaei G. Metabolic mediators of the effects of body-mass index, overweight, and obesity on coronary heart disease and stroke: a pooled analysis of 97 prospective cohorts with 1· 8 million participants. Elsevier; 2014.

15. Zhang H, Plutzky J, Skentzos S, Morrison F, Mar P, Shubina M, et al. Discontinuation of statins in routine care settings: a cohort study. Ann Intern Med. 2013;158(7):526-34. Epub 2013/04/03. doi: 10.7326/0003-4819-158-7-201304020-00004. PubMed PMID: 23546564; PubMed Central PMCID: PMCPMC3692286.

16. Alsheikh-Ali AA, Ambrose MS, Kuvin JT, Karas RH. The safety of rosuvastatin as used in common clinical practice: a postmarketing analysis. Circulation. 2005;111(23):3051-7. Epub 2005/05/25. doi: 10.1161/circulationaha.105.555482. PubMed PMID: 15911706.

17. Smith SL, Fischoff R, Klemp T. Medicare RBRVS 2011: The Physician's Guide. Chicago, IL: American Medical Association; 2011.

18. Russell LB, Ibuka Y, Carr D. How Much Time Do Patients Spend on Outpatient Visits?: The American Time Use Survey. The patient. 2008;1(3):211-22. Epub 2008/07/01. doi: 10.2165/1312067-200801030-00008. PubMed PMID: 22272927.
