# Supplementary material for: Cost-effectiveness of financial incentives and disincentives for improving food purchases and health through the US Supplemental Nutrition Assistance Program (SNAP): A microsimulation study
Source: PLoS Med. 2018 Oct 2;15(10):e1002661. doi: 10.1371/journal.pmed.1002661 (PMC6168180; doi:10.1371/journal.pmed.1002661)
Supplement: S10 Table — (DOCX) [file pmed.1002661.s011.docx]

# **S10 Table.** Results of Probabilistic Sensitivity Analyses at 5 Years and Lifetime. ^a^

|  | **Median Estimate (95% UI) ^g^** | |
| --- | --- | --- |
|  | **5 Years** | **Lifetime** |
| **F&V incentive (30%)** |  |  |
| Cases averted |  |  |
| Total CVD | 39,195 (25,695, 52,874) | 307,952 (198,768, 414,048) |
| Diabetes ^b^ | -361 (-387, -348) | -1,150 (-1,791, -407) |
| CVD deaths | 3,003 (1,765, 4,293) | 41,918 (26,321, 57,250) |
| QALYs gained | 19,276 (12,522, 27,518) | 661,026 (414,961, 917,319) |
| Healthcare cost-savings ($billion) ^c^ | 1.16 (1.59, 0.74) | 6.59 (8.98, 4.22) |
| Food subsidy cost ($billion) ^c^ | | |
| SNAP adults (age 35+ y) | 5.00 (3.48, 6.92) | 21.63 (15.06, 29.92) |
| All SNAP participants | 11.43 (7.83, 15.71) | 49.43 (33.86, 67.94) |
| ICER ($/QALY), by perspective ^d^ | | |
| Societal | Saving $1.10B ($1.51B, 0.80B) | Saving $6.27B ($8.55B, $4.02B) |
| Government affordability  (subsidizing SNAP adults age 35+) | 205,710 (113,715, 373,719) | 23,680 (12,440, 45,487) |
| Government affordability  (subsidizing all SNAP participants) | 541,191 (329,190, 927,765) | 66,680 (40,326, 116,376) |
| **F&V incentive/ SSB restriction** ^e^ | | |
| Cases averted |  |  |
| Total CVD | 94,062 (80,708, 107,384) | 799,644 (690,463, 911,432) |
| Diabetes | 29,908 (27,400, 32,145) | 170,134 (159,181, 181,144) |
| CVD deaths | 9,602 (8,442, 10,891) | 130,793 (114,456, 146,510) |
| QALYs gained | 46,000 (37,998, 55,328) | 2,101,013 (1,844,684, 2,399,494) |
| Healthcare cost-savings ($billion) ^c^ | 4.27 (4.74, 3.81) | 39.01 (42.61, 35.77) |
| Food subsidy cost ($billion) ^c^ | | |
| SNAP adults (age 35+ y) | 5.00 (3.48, 6.92) | 21.70 (15.10, 30.01) |
| All SNAP participants | 11.43 (7.83, 15.71) | 49.57 (33.97, 68.16) |
| ICER ($/QALY), by perspective ^d^ | | |
| Societal | Saving $4.07B ($4.52B, 3.73B) | Saving $37.13B ($40.57B, $34.05B) |
| Government affordability  (subsidizing SNAP adults age 35+) | 20,640 (Saving $0.54B, 63,665) | Saving $15.28B ($22.62B, ($6.95B) |
| Government affordability  (subsidizing all SNAP participants) | 160,280 (83,961, 262,899) | 6,106 (Saving 2.22B, 14,908) |
| **SNAP Plus (combined incentives/disincentives)** ^f^ | | |
| Cases averted |  |  |
| Total CVD | 116,772 (94,733, 138,206) | 939,290 (759,841, 1,115,086) |
| Diabetes | 25,649 (17,192, 34,869) | 143,831 (95,324, 199,838) |
| CVD deaths | 11,987 (9,486, 14,397) | 155,021 (122,679, 186,985) |
| QALYs gained | 55,634 (43,531, 68,148) | 2,450,454 (1,914,608, 2,970,611) |
| Healthcare cost-savings ($billion) ^c^ | 5.20 (6.28, 4.14) | 41.35 (51.56, 37.74) |
| Food subsidy cost ($billion) ^c^ |  |  |
| SNAP adults (age 35+ y) | -1.62 (-5.87, 2.06) | -7.21 (-25.72, 8.92) |
| All SNAP participants | -5.70 (-15.28, 2.91) | -25.02 (-67.10, 12.62) |
| ICER ($/QALY), by perspective ^d^ | | |
| Societal | Saving $4.95B ($5.98B, $4.19B) | Saving $39.36B ($49.09B, $30.22B) |
| Government affordability  (subsidizing SNAP adults age 35+) | Saving $6.62B ($11.20B, $2.53B) | Saving $46.82B ($69.47B, $26.76B) |
| Government affordability  (subsidizing all SNAP participants) | Saving $10.66B ($20.63B, $1.88B) | Saving $64.38B ($110.30B, $24.60B) |

**^a^** Health outcomes were evaluated among Supplemental Nutrition Assistance Program (SNAP) participants age 35-80 y at baseline, corresponding to 14.5 million adults in 2017 and followed until death or age 100, whichever first. Food subsidy costs were evaluated for adult SNAP participants only (N=14.6 million in 2017) and for all SNAP participants including children and adults age <35 y (N=42.1 million in 2017).[3,4]

^b^ Because we did not identify probable or convincing evidence of etiologic effects of fruits and vegetables on type 2 diabetes[[1](#_ENREF_8)] (see Table S3), the F&V incentive resulted in a slightly higher number of diabetes cases due to increased overall survival from prevented CVD.

^c^ All costs were inflated to constant 2017 US dollars using the Bureau of Labor Statistics’ Consumer Price Index.[2] Costs and quality-adjusted life years (QALYs) were discounted by 3% annually. Healthcare cost-savings were calculated as averted direct costs from chronic/acute disease states, surgical procedures, screening and drug use; and here are shown after subtraction of the intervention policy administrative costs.

^d^ Incremental cost-effectiveness ratio (ICER) thresholds were evaluated at $150,000/QALY and $50,000/QALY from three perspectives including (1) societal, (2) governmental affordability including subsidy costs for SNAP adults age 35+ y, and (3) government affordability including subsidy costs for all SNAP participants including children and adults age <35 y. As appropriate, the societal perspective did not include food subsidy costs or disincentive gains because these represent a transfer (like a tax or tax break) from one segment of society to another. Additional potential health benefits and healthcare cost-savings from these dietary interventions were conservatively excluded, including potential benefits for cancer in adults as well as all potential health benefits in children and young adults age <35 y.

^e^ Assuming that with full restriction, 50% of SSB purchases in retail venues shift from SNAP dollars to participants’ other food dollars.

^f^ A 30% financial incentive for fruits, vegetables, nuts, whole grains, fish, and plant-based oils; and a 30% disincentive for sugar-sweetened beverages (SSBs), junk food, and processed meats.

^g^ The values from probabilistic sensitivity analyses are presented as the median and 95% UI for each scenario based on 1,000 iterations. These models jointly incorporated the uncertainties in policy effect sizes, diet-disease relative risks including their variations by age, CVD risks, implementation costs, food unit costs, formal and informal health care cost, and utility weights (see S9 Table). The full results of each 1,000 simulations are shown in Fig 5.

**References**

1. Micha R, Shulkin ML, Penalvo JL, Khatibzadeh S, Singh GM, Rao M, et al. Etiologic effects and optimal intakes of foods and nutrients for risk of cardiovascular diseases and diabetes: Systematic reviews and meta-analyses from the Nutrition and Chronic Diseases Expert Group (NutriCoDE). PLoS One. 2017;12(4):e0175149. Epub 2017/04/28. doi: 10.1371/journal.pone.0175149. PubMed PMID: 28448503; PubMed Central PMCID: PMCPMC5407851.

2. U.S. Department of Labor. The Bureau of Labor Statistics' Consumer Price Index 2017 [cited 2017 October 16]. Available from: <https://www.bls.gov/data/#prices>.

3. U.S. Department of Agriculture. Supplemental Nutrition Assistance Program Participation and Costs 2018 [cited 2018 Jan 24]. Available from: <https://fns-prod.azureedge.net/sites/default/files/pd/SNAPsummary.pdf>.

4. U.S. Department of Agriculture. Characteristics of Supplemental Nutrition Assistance Program Households: Fiscal Year 2016 2017 [cited 2018 Jan 24]. Available from: <https://fns-prod.azureedge.net/sites/default/files/ops/Characteristics2016.pdf>.
