# Supplementary material for: Cost-effectiveness of financial incentives and disincentives for improving food purchases and health through the US Supplemental Nutrition Assistance Program (SNAP): A microsimulation study
Source: PLoS Med. 2018 Oct 2;15(10):e1002661. doi: 10.1371/journal.pmed.1002661 (PMC6168180; doi:10.1371/journal.pmed.1002661)
Supplement: S11 Table — (DOCX) [file pmed.1002661.s012.docx]

# **S11 Table.** Comparison of Relative Risks for CHD Observed in Prospective Cohort Studies of Dietary Patterns and Estimated Relative Risks for Individual Dietary Factors. ^a^

| **Study** | **Estimate type** | **Q2** | **Q3** | | **Q4** | | **Q5** | **Average of all quintiles** ^b^ | **Mean absolute risk difference, calculated vs. observed** ^c^ |
| --- | --- | --- | --- | --- | --- | --- | --- | --- | --- |
| Health Professionals Study – Prudent Dietary Pattern[[1](#_ENREF_9)] | Calculated RR in each quintile ^d^ | 0.86 | 0.82 | | 0.74 | | 0.63 | 0.75 | 0.07 |
|  | Calculated RR, adjusted for time ^e^ | 0.88 | 0.83 | | 0.76 | | 0.66 | 0.78 | 0.04 |
|  | Observed RR | 0.90 | 0.83 | | 0.79 | | 0.75 | 0.82 |  |
|  |  |  |  | |  | |  |  |  |
| Health Professionals Study – Western Dietary Pattern[[1](#_ENREF_9)] | Calculated RR in each quintile ^d^ | 1.12 | 1.16 | | 1.19 | | 1.30 | 1.19 | -0.10 |
|  | Calculated RR, adjusted for time ^e^ | 1.10 | 1.14 | | 1.17 | | 1.26 | 1.17 | -0.12 |
|  | Observed RR | 1.21 | 1.27 | | 1.27 | | 1.43 | 1.29 |  |
|  |  |  |  | |  | |  |  |  |
| Nurses’ Health Study – Prudent Dietary Pattern[2] | Calculated RR in each quintile ^d^ | 0.91 | 0.80 | | 0.75 | | 0.66 | 0.77 | 0.05 |
|  | Calculated RR, adjusted for time ^e^ | 0.92 | 0.82 | | 0.77 | | 0.69 | 0.80 | 0.02 |
|  | Observed RR | 0.95 | 0.83 | | 0.76 | | 0.76 | 0.82 |  |
|  |  |  |  | |  | |  |  |  |
| Nurses’ Health Study – Western Dietary Pattern [2] | Calculated RR in each quintile ^d^ | 1.04 | 1.07 | | 1.15 | | 1.23 | 1.12 | -0.08 |
|  | Calculated RR, adjusted for time ^e^ | 1.03 | 1.06 | | 1.13 | | 1.20 | 1.10 | -0.10 |
|  | Observed RR | 1.01 | 1.10 | | 1.26 | | 1.46 | 1.20 |  |
|  |  |  |  | |  | |  |  |  |
| Nurses’ Health Study – Mediterranean Dietary Pattern[3] | Calculated RR in each quintile ^f^ | 0.93 | 0.82 | | 0.76 | | 0.72 | 0.80 | 0.04 |
|  | Calculated RR, adjusted for time ^e^ | 0.93 | 0.83 | | 0.77 | | 0.73 | 0.81 | 0.03 |
|  | Observed RR | 0.92 | 0.87 | | 0.87 | | 0.71 | 0.84 |  |
|  |  | Men | | Women | | Overall | |  |  |
| EPIC Greece – Mediterranean Dietary Pattern[4] | Calculated RR per 2 unit increase ^g^ | 0.90 | | 0.91 | | 0.90 | |  | -0.12 |
|  | Observed RR | 0.81 | | 0.75 | | 0.78 | |  |  |
|  |  |  | | |  | | |  |  |
| SUN Cohort Spain – Mediterranean Dietary Pattern[5] | Calculated RR per 2 unit increase ^h^ | 0.75 | | | | | |  | -0.01 |
|  | Observed RR | 0.74 | | | | | |  |  |
|  |  | | | | | | |  |  |

^a^ The observed multivariable-adjusted relative risk (RR) in each category or per each unit of the dietary pattern was compared to the predicted effect calculated by combining the reported differences in individual dietary factors (including fruit, vegetables, whole grains, fish, processed meat, *trans* fat, polyunsaturated fat) across each category or per unit of the diet pattern with their Nutrition and Chronic Diseases Expert Group (NutriCoDE)-estimated individual quantitative effects, assuming a multiplicative relation between RRs for individual components. We primarily utilized the RRs for foods and excluded overlapping components (e.g., whole grains and fiber; or meats and saturated fats) in these analyses. The calculated RRs also assumed no benefits from changes in other dietary factors (e.g., coffee) for which we had not determined a causal etiologic effect, which could cause the observed RRs to be greater than the calculated RRs; and also incorporated the NutriCoDE threshold of optimal intake, beyond which no further benefit was assumed.

*^b^* Based on the mean of beta-coefficients (ln RR’s) across quintiles within each study.

*^c^* Based on the mean absolute risk difference of calculated vs. observed RR’s [(1-calculated RR) - (1-observed RR)]. Compared to observed RRs as the reference, positive values represent overestimation of calculated RRs, while negative values represent underestimation of calculated RRs.

*^d^* Dietary factors reported and included were fruits, vegetables, whole grains, fish, processed meat, *trans* fat, and polyunsaturated fat.

*^e^* Accounting for observed declining dietary differences over time in the dietary pattern studies in these specific cohorts.

*^f^* Dietary factors reported and included were fruit, vegetables, whole grains, omega-3s, processed and red meat, and *trans* fat.

*^g^* Dietary factors reported and included were vegetables plus legumes, fruits and nuts, fish, processed meat, and polyunsaturated fat. The dietary comparisons used were for the 75th vs. 25th percentiles.

^h^ Dietary factors reported and included were vegetables plus legumes, fruits, fish, whole grains, nuts, processed meats, and polyunsaturated fat.

Table adapted with permission from Micha R, Shulkin ML, Penalvo JL, et al. Etiologic effects and optimal intakes of foods and nutrients for risk of cardiovascular diseases and diabetes: Systematic reviews and meta-analyses from the Nutrition and Chronic Diseases Expert Group (NutriCoDE). *PLoS One*. 2017;12(4):e0175149.

**References**

1. Hu FB, Rimm EB, Stampfer MJ, Ascherio A, Spiegelman D, Willett WC. Prospective study of major dietary patterns and risk of coronary heart disease in men. Am J Clin Nutr. 2000;72(4):912-21. PubMed PMID: 11010931.

2. Fung TT, Willett WC, Stampfer MJ, Manson JE, Hu FB. Dietary patterns and the risk of coronary heart disease in women. Archives of internal medicine. 2001;161(15):1857-62. PubMed PMID: 11493127.

3. Fung TT, Rexrode KM, Mantzoros CS, Manson JE, Willett WC, Hu FB. Mediterranean diet and incidence of and mortality from coronary heart disease and stroke in women. Circulation. 2009;119(8):1093-100. Epub 2009/02/18. doi: 10.1161/circulationaha.108.816736. PubMed PMID: 19221219; PubMed Central PMCID: PMCPMC2724471.

4. Trichopoulou A, Bamia C, Trichopoulos D. Anatomy of health effects of Mediterranean diet: Greek EPIC prospective cohort study. BMJ (Clinical research ed). 2009;338:b2337. Epub 2009/06/25. doi: 10.1136/bmj.b2337. PubMed PMID: 19549997; PubMed Central PMCID: PMCPMC3272659.

5. Martinez-Gonzalez MA, de la Fuente-Arrillaga C, Lopez-Del-Burgo C, Vazquez-Ruiz Z, Benito S, Ruiz-Canela M. Low consumption of fruit and vegetables and risk of chronic disease: a review of the epidemiological evidence and temporal trends among Spanish graduates. Public Health Nutr. 2011;14(12A):2309-15. Epub 2011/12/15. doi: 10.1017/s1368980011002564. PubMed PMID: 22166189.
