# Supplementary material for: Cost-effectiveness of financial incentives and disincentives for improving food purchases and health through the US Supplemental Nutrition Assistance Program (SNAP): A microsimulation study
Source: PLoS Med. 2018 Oct 2;15(10):e1002661. doi: 10.1371/journal.pmed.1002661 (PMC6168180; doi:10.1371/journal.pmed.1002661)
Supplement: S12 Table — (DOCX) [file pmed.1002661.s013.docx]

# **S12 Table**. Comparison of Relative Risks for CHD Calculated Based on Changes in Systolic Blood Pressure and LDL-cholesterol in Randomized Controlled Feeding Trials of Dietary Patterns vs. Estimated Relative Risks for Individual Dietary Factors.

| **Dietary risk factor** | **Change in SBP (mmHg) in dietary feeding RCTs** *^1^* | **Change in LDL-C (mg/dL) in dietary feeding RCTs** *^1^* | **Predicted RR of CHD in dietary feeding RCTs, based on SBP effect** *^2^* | **Predicted RR of CHD in dietary feeding RCTs, based on LDL-C effect** *^2^* | **Multiplicative RR of CHD per serving in dietary feeding RCTs, based on joint SBP and LDL-C effects** | **Estimated RR of CHD from cohort studies**  **(NutriCoDE relative risks, see Table 2)** |
| --- | --- | --- | --- | --- | --- | --- |
| Fruits, per serving/d  (100 g/d) | -0.33 | -1.5 | 0.99 | 0.94 | 0.93 | 0.94  (0.91, 0.98) |
| Vegetables, per serving/d  (100 g/d) | -0.18 | -1.6 | 0.99 | 0.94 | 0.93 | 0.95  (0.92, 0.98) |
| Nuts/seeds, per serving/wk  (28.35 g) | -0.92 | -1 | 0.97 | 0.96 | 0.93 | 0.93  (0.91, 0.96) |
| Whole grains, per serving/d  (50 g/d) | -0.11 | -3.2 | 1.00 | 0.88 | 0.88 | 0.97  (0.94, 0.99) |
| Fish, per serving/d  (100 g/d) | N/A*^3^* | -3.4 | N/A *^3^* | 0.87 | 0.87 | 0.66  (0.50, 0.87) |
| Red meat, per serving/d  (100 g/d) | 3.20 | 1.1 | 1.12 | 1.04 | 1.17 | 1.17  (1.05, 1.30) *^4^* |
| Dietary fiber, per 20 g/d  (20 g/d) *^5^* | -3.00 | -3.9 | 0.89 | 0.86 | 0.77 | 0.76  (0.68, 0.85) |

*^1^* For systolic blood pressure (SBP), trials include OmniHeart (protein diet vs. baseline diet, carbohydrate diet vs. baseline diet, and unsaturated fat diet vs. baseline diet),[5] DASH-sodium (high sodium DASH diet vs. high sodium control diet),[4] and DASH (combination diet vs. control diet, fruit and vegetable diet vs. control diet).[3] For LDL-C, trials include OmniHeart (protein diet vs. baseline diet, carbohydrate diet vs. baseline diet, and unsaturated fat diet vs. baseline diet), DASH-sodium (high sodium DASH diet vs. high sodium control diet, intermediate sodium DASH diet vs. intermediate sodium control diet, low sodium DASH diet vs. low sodium control diet). Results reflect pooled meta-regression models simultaneously accounting for all dietary changes in these dietary patterns trials, i.e. changes in each dietary factor in this Table.

*^2^* Based on the observed association between SBP and incident coronary heart disease (CHD) events and LDL-C and incident CHD events in large pooling projects of prospective cohort studies.[[1](#_ENREF_9),2,6]

*^3^* Analysis of the impact of including fish in the blood pressure meta-regression model indicated that small changes in fish intake caused improbably large changes in blood pressure. Therefore, fish was not included in the blood pressure meta-regression.

*^4^* Based on prospective cohort studies, we identified evidence for an etiologic relative risk (RR) for CHD for processed meat, but not unprocessed red meat. Because these feeding studies evaluated only total meat consumption, the corresponding RR for cohort studies represents the estimated RR for total meat consumption, based on approximately 25% of total meat consumption being processed meat, a 100 g serving size, and assuming no significant etiologic effect of unprocessed red meat.

*^5^* Due to their substantial overlap, dietary fiber was excluded from meta-regression models estimating changes in SBP and LDL-C in which fruits, vegetables, nuts and seeds, and whole grains were independent variables. Likewise, fruits, vegetables, nuts and seeds, and whole grains were excluded in models in which dietary fiber was an independent variable.

Table adapted with permission from Micha R, Shulkin ML, Penalvo JL, et al. Etiologic effects and optimal intakes of foods and nutrients for risk of cardiovascular diseases and diabetes: Systematic reviews and meta-analyses from the Nutrition and Chronic Diseases Expert Group (NutriCoDE). *PLoS One*. 2017;12(4):e0175149.

**References**

1. Hu FB, Rimm EB, Stampfer MJ, Ascherio A, Spiegelman D, Willett WC. Prospective study of major dietary patterns and risk of coronary heart disease in men. Am J Clin Nutr. 2000;72(4):912-21. PubMed PMID: 11010931.

2. Fung TT, Willett WC, Stampfer MJ, Manson JE, Hu FB. Dietary patterns and the risk of coronary heart disease in women. Archives of internal medicine. 2001;161(15):1857-62. PubMed PMID: 11493127.

3. Appel LJ, Moore TJ, Obarzanek E, Vollmer WM, Svetkey LP, Sacks FM, et al. A clinical trial of the effects of dietary patterns on blood pressure. DASH Collaborative Research Group. N Engl J Med. 1997;336(16):1117-24. Epub 1997/04/17. doi: 10.1056/nejm199704173361601. PubMed PMID: 9099655.

4. Sacks FM, Svetkey LP, Vollmer WM, Appel LJ, Bray GA, Harsha D, et al. Effects on blood pressure of reduced dietary sodium and the Dietary Approaches to Stop Hypertension (DASH) diet. DASH-Sodium Collaborative Research Group. N Engl J Med. 2001;344(1):3-10. PubMed PMID: 11136953.

5. Appel LJ, Sacks FM, Carey VJ, Obarzanek E, Swain JF, Miller ER, 3rd, et al. Effects of protein, monounsaturated fat, and carbohydrate intake on blood pressure and serum lipids: results of the OmniHeart randomized trial. JAMA. 2005;294(19):2455-64. PubMed PMID: 16287956.

6. He FJ, MacGregor GA. Effect of modest salt reduction on blood pressure: a meta-analysis of randomized trials. Implications for public health. Journal of human hypertension. 2002;16(11):761-70. PubMed PMID: 12444537.
