# Supplementary material for: Cost-effectiveness of financial incentives and disincentives for improving food purchases and health through the US Supplemental Nutrition Assistance Program (SNAP): A microsimulation study
Source: PLoS Med. 2018 Oct 2;15(10):e1002661. doi: 10.1371/journal.pmed.1002661 (PMC6168180; doi:10.1371/journal.pmed.1002661)
Supplement: S13 Table — (DOCX) [file pmed.1002661.s014.docx]

# **S13 Table**. Comparison of Relative Risks for CHD Observed in a Large Randomized Clinical Trial of Dietary Patterns vs. Estimated Relative Risks for Individual Dietary Factor. *^1^*

| **Dietary Factor** | **Achieved change in the EVOO group, g/d** *^2^* | **Achieved change in the nut group, g/d** | **NutriCoDE -estimated effect on MI[2]** | **NutriCoDE serving size, g/d** | **Calculated effect on MI in the EVOO group[2]** | **Calculated effect on MI in the nut group[2]** | **Calculated effect on MI in combined groups[2]** |
| --- | --- | --- | --- | --- | --- | --- | --- |
| Fruits | 6.25 | 12.5 | 0.94 | 100 | 0.996 | 0.992 | 0.994 |
| Vegetables | 1.75 | 10.0 | 0.95 | 100 | 0.999 | 0.995 | 0.997 |
| Beans/legumes | 2.40 | 2.4 | 0.77 | 100 | 0.994 | 0.994 | 0.994 |
| Nuts/seeds | 3.25 | 21.0 | 0.77 *^3^* | 16.2 | 0.949 | 0.713 | 0.822 |
| Seafood n-3 fatty acids | 0.11 | 0.12 | 0.92 *^4^* | 0.1 | 0.912 | 0.905 | 0.909 |
| Extra-virgin olive oil (%E) | 4.97 | 1.08 | 0.90 *^5^* | 5.0 | 0.901 | 0.977 | 0.938 |
|  | ***Calculated Overall Effect (all six dietary factors)*** | | | | ***0.771*** | ***0.618*** | ***0.691*** |
|  | ***Observed Effect in PREDIMED*** | | | | ***0.800*** | ***0.740*** | ***0.770*** |

*^1^* For consistency with the other validity analyses (S4-S5 Tables), we focused on results for CHD in the PREDIMED (Prevencion con Dieta Mediterranea) trial.[3] A similar analysis was previously reported using 2010 estimated RR’s;[4] the findings here are based on the updated RR’s in Micha et al.[1]

*^2^* Values are g/d except for extra virgin olive oil (EVOO), which is percent energy (%E).

*^3^* Assuming half of myocardial infarctions (MIs) were fatal, and half nonfatal.

*^4^* Assuming half of MI’s were fatal, as the risk reduction for marine n-3 fatty acids is specific for fatal MI, not nonfatal MI.

*^5^* Assuming effects on MI are similar to those of vegetable oil polyunsaturated fatty acids.

EVOO, extra-virgin olive oil. NutriCoDE, Nutrition and Chronic Diseases Expert Group.

Table adapted with permission from Micha R, Shulkin ML, Penalvo JL, et al. Etiologic effects and optimal intakes of foods and nutrients for risk of cardiovascular diseases and diabetes: Systematic reviews and meta-analyses from the Nutrition and Chronic Diseases Expert Group (NutriCoDE). *PLoS One*. 2017;12(4):e0175149.

**References**

1. Micha R, Shulkin ML, Penalvo JL, Khatibzadeh S, Singh GM, Rao M, et al. Etiologic effects and optimal intakes of foods and nutrients for risk of cardiovascular diseases and diabetes: Systematic reviews and meta-analyses from the Nutrition and Chronic Diseases Expert Group (NutriCoDE). PLoS One. 2017;12(4):e0175149. Epub 2017/04/28. doi: .1371/journal.pone.0175149. PubMed PMID: 28448503; PubMed Central PMCID: PMCPMC5407851.

2. Martinez-Gonzalez MA, de la Fuente-Arrillaga C, Lopez-Del-Burgo C, Vazquez-Ruiz Z, Benito S, Ruiz-Canela M. Low consumption of fruit and vegetables and risk of chronic disease: a review of the epidemiological evidence and temporal trends among Spanish graduates. Public Health Nutr. 2011;14(12A):2309-15. Epub 2011/12/15. doi: 10.1017/s1368980011002564. PubMed PMID: 22166189.

3. Estruch R, Ros E, Salas-Salvado J, Covas MI, Corella D, Aros F, et al. Primary prevention of cardiovascular disease with a Mediterranean diet. The New England journal of medicine. 2013;368(14):1279-90. Epub 2013/02/26. doi: 10.1056/NEJMoa1200303. PubMed PMID: 23432189.

4. Mozaffarian D. Mediterranean diet for primary prevention of cardiovascular disease. The New England journal of medicine. 2013;369(7):673-4. Epub 2013/08/16. doi: 10.1056/NEJMc1306659#SA3. PubMed PMID: 23944310.
