# Supplementary material for: Cost-effectiveness of financial incentives and disincentives for improving food purchases and health through the US Supplemental Nutrition Assistance Program (SNAP): A microsimulation study
Source: PLoS Med. 2018 Oct 2;15(10):e1002661. doi: 10.1371/journal.pmed.1002661 (PMC6168180; doi:10.1371/journal.pmed.1002661)
Supplement: S1 Text — (DOCX) [file pmed.1002661.s015.docx]

# **S1 Text. CVD-PREDICT Microsimulation Model**

## ***Model Description***

The CVD Predict model simulates aging and health transitions of individual adults using a variety CVD-related epidemiological data, including adapted Framingham-based risk scores for ischemic heart disease and stroke, calibrated and validated to observed risk in the US adult population. The model uses a micro-simulation approach to more accurately model the natural history of CVD. Specifically, the ability to store and modify specific CVD risk factors on an individual basis and update individual-level CVD disease history are great strengths of the model. Full details of the CVD Predict Model and validation result has been published elsewhere.[[1](#_ENREF_1)]

Briefly, the CVD PREDICT model is populated with a database of individual adults age 35+ years with accompanying risk factor data. The CVD risk factors used to predict risk of disease transitions include age, sex, systolic blood pressure, total cholesterol, HDL cholesterol, smoking status, and diabetes status. The model also considers an individual’s prior history of having a CVD event and populates these individuals in their respective CVD health states at the start of each 1-year model run. To achieve national representativeness, adult participants from the National Health and Nutrition Examination Surveys (NHANES) are sampled with replacement using NHANES sample weights to create a representative population of 1,000,000 individuals. Risk factor and dietary distributions are therefore directly embedded in the model population based on NHANES reports all of the variables at baseline. Descriptive statistics for the model population (Table 1) were generated using the statistical software packages STATA 14.

In each yearly time frame, three main updates occur: updating of the risk factors (e.g. an increase in systolic blood pressure), potential transitions into a different CVD health state , and preventive interventions (e.g. screening, medication, lifestyle modifications, or as in this case, food and nutrition policy intervention). These annual simulations are repeated for the duration of the time period of interest (e.g., 5 years, 10 years, 20 years, lifetime), with a necessarily closed population design to evaluate lifetime risk and an open population design (new 35 year-olds entering the population each year) for other discrete time periods.

For each intervention scenario as well as no new intervention (natural history), the model generates the predicted changes in probability of each health outcome at the individual level, both overall and stratified by age (35-44, 45-54, 55-64, 65-74, 75+), sex (male, female), race (non-Hispanic white, non-Hispanic black, Hispanic, other), and education (< high school, high school graduate/some college, college graduate or above) and health insurance status (Medicaid, Medicare, others). The model incorporates a conservation population growth rate estimate for each incoming cohort, added to the model population annually. The addition of new, annual cohorts further allows the model to account for secular trends in risk factor and demographic data*.*

Health states, events, and costs are then determined based on each yearly population simulation. The CVD PREDICT model tracks each cardiometabolic event (CVD, diabetes) for every individual run through the model, including the number of deaths arising from each type of event, the average life expectancy of those with and without CVD, the number of individuals who have ever had an event, and the yearly prevalence of every disease state. Additionally, as costs and health state utilities are accrued by each individual in every (yearly) model cycle, the model also produces event-associated and overall healthcare costs, and quality-adjusted life years (QALYs) which capture both number and quality of years survived by each individual. Utility values ranging between 0 (death) and 1 (perfect health) were assigned to each health state (disease free, cardiac arrest, myocardial infarction, angina, stroke, death) based on EuroQOL 5 Dimensions (EQ-5D) questionnaire results from the Medical Expenditure Panel Survey (MEPS).[[2](#_ENREF_2)] Population totals and averages are calculated and stored once the appropriate number of individuals complete simulation over the relevant time period.

Several methodological aspects of CVD-PREDICT are new compared to prior publications. First, the 5, 10, and 20 year time horizons are open cohorts that bring in new individuals reflective of US demographics each year, allowing assessment of not only cost-effectiveness for a static cohort aging through the model, but also the financial impact of the intervention that is consistent with changing US demographics and population aging. Second, we present data stratified by health insurance status to inform the potential differential impact of the policy on various healthcare payers. Third, we provide additional data incorporating productivity gains and losses using US data to capture a societal perspective

## ***Model Validation***

We have previously calibrated and validated our micro-simulation model using recent NHANES data with a population from 1999-2011.[[3](#_ENREF_3)] Model-based (simulated) results were compared to observed all-cause and CVD specific mortality for the same starting population using survival curves and receiver operating characteristic curves (Available from <http://journals.sagepub.com/doi/abs/10.1177/0272989X17706081?journalCode=mdma>).  Five-year and 10-year CVD and all-cause mortality from the CVD PREDICT model fell within the 95% CIs of the observed data. For example, observed 10-year all-cause mortality in NHANES v. the simulation model was 11.2% (95% CI, 10.3% to 12.2%) v. 10.9%; corresponding results for CVD mortality were 2.2% (1.8% to 2.7%) v. 2.6%. CVD PREDICT model-based all-cause mortality projections at 20 and 30 years were 27.7% and 47.8%; the corresponding results for the life table extrapolation of the same starting population were 28.1% and 48.9%, respectively. Life expectancy was 82.5 years and 81.7 years for the CVD PREDICT model-based and life table-based projections, respectively. Areas under the ROC curves for model-predicted 10-year all-cause and CVD mortality risks were 0.83 (0.81 to 0.85) and 0.84 (0.81 to 0.88), respectively; corresponding results for 5-year risks were 0.80 (0.77 to 0.83) and 0.81 (0.75 to 0.87), respectively.

Because the predictive risk functions that determine the model risk for CVD (Framingham risk score, AHA/ACC pooled cohort ASCVD risk score) were developed primarily for adults age 35 to 79 years, the model was not extended to younger ages, which could reduce validity. Notably, because very few events occur before the age of 35 years (0.6% among those 20-39 compared with 5-30% in older age groups), excluding very young adults would have little influence on findings. Further, due to the dynamic (open cohort) nature of the CVD-PREDICT model, younger people do enter the model over time. For example, over a 20 year time period, American adults aged 20 years at the start of intervention enter into the model at year 15, when they turn 35 years (and when their risk for CVD starts to become meaningful). Importantly, the costs and cost-effectiveness analyses incorporate the cost of the intervention for everyone on SNAP (children, young adults, older adults), while the estimated healthy benefits are only calculated for adults who are 35 years and older. Because healthier diets are likely to produce health benefits at younger ages, the overall health benefits and cost-effectiveness are likely to be underestimated (conservative).

**References**

1. Pandya A, Sy S, Cho S, Alam S, Weinstein MC, Gaziano TA. Validation of a Cardiovascular Disease Policy Micro-Simulation Model using Both Survival and Receiver Operating Characteristic Curves. Medical Decision Making. 2017:0272989X17706081.

2. Sullivan PW, Ghushchyan V. Preference-based EQ-5D index scores for chronic conditions in the United States. Medical Decision Making. 2006;26(4):410-20.

3. Pandya A, Sy S, Cho S, Alam S, Weinstein MC, Gaziano TA. Validation of a Cardiovascular Disease Policy Microsimulation Model Using Both Survival and Receiver Operating Characteristic Curves. Med Decis Making. 2017;37(7):802-14. Epub 2017/05/12. doi: 10.1177/0272989X17706081. PubMed PMID: 28490271; PubMed Central PMCID: PMCPMC5577377.
