# Supplementary material for: Cost-effectiveness of financial incentives and disincentives for improving food purchases and health through the US Supplemental Nutrition Assistance Program (SNAP): A microsimulation study
Source: PLoS Med. 2018 Oct 2;15(10):e1002661. doi: 10.1371/journal.pmed.1002661 (PMC6168180; doi:10.1371/journal.pmed.1002661)
Supplement: S2 Text — (DOCX) [file pmed.1002661.s016.docx]

# **S2 Text. Changes Over Time in SNAP Participation**

Many SNAP participants may not remain eligible or choose to continue participating in SNAP over time. Acknowledging that varying proportions of SNAP participants shift into and out of the population over time, one salient issue for our modeling is how quickly health benefits may accrue from a change in diet. If benefits accrue within a reasonable period, then if one participant remains on SNAP for 2 years and experiences lower risk from the intervention within these two years; and subsequently is replaced by a different SNAP participant who joins the program and then experiences their own reduced risk from the intervention; then the summed health effect will be broadly similar whether these are the same person or two different people with similar demographics and risk characteristics. Randomized controlled feeding trials demonstrate significant reductions in cardiometabolic risk factors within 30 days of improved dietary composition (without changes in calorie consumption or weight) [[1-](#_ENREF_4)3]; while the PREDIMED interventional study demonstrated a separation of risk curves for clinical endpoints within months of changes in diet.[[4](#_ENREF_7)] Thus, it is reasonable to estimate that changes in risk occur within the same year of the intervention for each participant. In other words, a person’s probability of cardiometabolic risk will be lower from the intervention during the year they are participating, whether or not they rotate off SNAP in future years.

A second issue is how the total numbers of SNAP participants and their demographics, including prevalence of various health states, may change over time. It is recognized that absolute numbers of SNAP participants in the US can change significantly over time, for example depending on shifts in the national economy. Demographics may also change as both US demographics and relative participation shift. In a model of future risk, it would be difficult to accurately predict how absolute numbers of SNAP participants, and to a lesser extent their demographics, may shift over 20 years or a lifetime of current participants. Thus, our model assumes that shifts in SNAP will follow the general demographic shifts in the US population, already accounted for in the model. If absolute SNAP participation meaningfully increases over time, then absolute benefits and costs of the intervention will be higher; and if participation meaningfully decreases, then absolute benefits and costs will be lower. However, regardless of demographic shifts or even changes in absolute numbers, the main comparisons of incremental cost-effectiveness (ICER= costs/QALY) will remain relatively stable, as these are (a) a ratio of costs to benefits within the population and (b) determined by comparing the incremental effects of each intervention scenario to a “status quo” scenario. The latter point is especially relevant as any potential absolute participation or demographic shifts will occur equally in both scenarios, greatly reducing any impact on the incremental per person costs, health benefits, or cost-effectiveness. For each of these reasons, the findings for the incremental comparative and cost-effectiveness of the different interventions in our investigation are relatively robust to changes in SNAP participants over time.

**References**

1. Svetkey LP, Simons-Morton D, Vollmer WM, Appel LJ, Conlin PR, Ryan DH, et al. Effects of dietary patterns on blood pressure: subgroup analysis of the Dietary Approaches to Stop Hypertension (DASH) randomized clinical trial. Arch Intern Med. 1999;159(3):285-93. PubMed PMID: 9989541.

2. Sacks FM, Svetkey LP, Vollmer WM, Appel LJ, Bray GA, Harsha D, et al. Effects on blood pressure of reduced dietary sodium and the Dietary Approaches to Stop Hypertension (DASH) diet. DASH-Sodium Collaborative Research Group. N Engl J Med. 2001;344(1):3-10. doi: 10.1056/NEJM200101043440101. PubMed PMID: 11136953.

3. Appel LJ, Sacks FM, Carey VJ, Obarzanek E, Swain JF, Miller ER, 3rd, et al. Effects of protein, monounsaturated fat, and carbohydrate intake on blood pressure and serum lipids: results of the OmniHeart randomized trial. JAMA. 2005;294(19):2455-64. doi: 10.1001/jama.294.19.2455. PubMed PMID: 16287956.

4. Estruch R, Ros E, Salas-Salvado J, Covas MI, Corella D, Aros F, et al. Primary Prevention of Cardiovascular Disease with a Mediterranean Diet Supplemented with Extra-Virgin Olive Oil or Nuts. N Engl J Med. 2018;378(25):e34. Epub 2018/06/14. doi: 10.1056/NEJMoa1800389. PubMed PMID: 29897866.
