# Supplementary material for: Cost-effectiveness of financial incentives and disincentives for improving food purchases and health through the US Supplemental Nutrition Assistance Program (SNAP): A microsimulation study
Source: PLoS Med. 2018 Oct 2;15(10):e1002661. doi: 10.1371/journal.pmed.1002661 (PMC6168180; doi:10.1371/journal.pmed.1002661)
Supplement: S3 Text — (DOCX) [file pmed.1002661.s017.docx]

# **S3 Text. Shifts in Food Purchases between SNAP vs. non-SNAP Dollars**

In a purely rational economic model, SNAP participants with discretionary spending may shift their food spending between SNAP vs. non-SNAP Dollars due to financial incentives or disincentives. The extent of potential shifting will vary across people depending on a range of factors, including but not limited to differences in income. Furthermore, such economic stimuli may alter not only the main targeted food item but also dietary complements and substitutes.

We addressed these possibilities in several ways. First, in terms of shifting between SNAP vs. non-SNAP dollars, the randomized HIP trial[[1](#_ENREF_7)] that provided F&V incentives to SNAP participants identified very little actual shifting in spending (almost zero), far less than predicted based on economic theory of individual rational actors. In addition, because the main effect measure from HIP used in our model was the effect of the SNAP subsidy on total purchases of fruits and vegetables (from supermarkets and other venues accepting EBT cards, as in our analysis) using all participant dollars from both SNAP and non-SNAP sources, the intervention effect size already incorporates the average shifting of spending using SNAP vs. other dollars.

Nonetheless, we incorporated an estimate of potential shifting in both our main and sensitivity analyses, which would cause the dietary and health effects of the intervention to be smaller for the same costs (i.e., reduced cost-effectiveness). After much consideration, we elected not to model these potential shifts between SNAP vs. non-SNAP dollars differently for different income levels, based on little empiric evidence to make such assumptions and also based on the relatively small potential impact on the overall results if we utilized differential shifts in different people vs. one common average shift for the whole population.

**References**

1. Svetkey LP, Simons-Morton D, Vollmer WM, Appel LJ, Conlin PR, Ryan DH, et al. Effects of dietary patterns on blood pressure: subgroup analysis of the Dietary Approaches to Stop Hypertension (DASH) randomized clinical trial. Arch Intern Med. 1999;159(3):285-93. PubMed PMID: 9989541.
