# Supplementary material for: Cost-effectiveness of financial incentives and disincentives for improving food purchases and health through the US Supplemental Nutrition Assistance Program (SNAP): A microsimulation study
Source: PLoS Med. 2018 Oct 2;15(10):e1002661. doi: 10.1371/journal.pmed.1002661 (PMC6168180; doi:10.1371/journal.pmed.1002661)
Supplement: S4 Text — (DOCX) [file pmed.1002661.s018.docx]

# **S4 Text: Assessment of Validity and Bias in Diet-Disease Etiologic Effects [**adapted with permission from Micha R, Shulkin ML, Penalvo JL, et al. Etiologic effects and optimal intakes of foods and nutrients for risk of cardiovascular diseases and diabetes: Systematic reviews and meta-analyses from the Nutrition and Chronic Diseases Expert Group (NutriCoDE). *PLoS One*. 2017;12(4):e0175149.]

While nearly all identified observational studies in the utilized meta-analyses utilized multivariable adjustment for major demographics, CVD risk factors and, in many cases, other dietary factors,[[1](#_ENREF_8)] we recognized that clustering of dietary patterns could still cause unmeasured confounding, e.g., from clustering of healthful factors such as fruits, vegetables, and whole grains as well as inverse correlations of these with harmful factors such as SSBs or processed meats. Thus, even with multi-variable adjustment, our final calculated etiologic effects from studies of an individual dietary component might overestimate its effects, as compared with the true effect when the dietary component is consumed as part of an overall diet pattern.

To assess potential bias from dietary pattern effects, we performed 3 validity analyses including based on: (a) prospective long-term observational studies evaluating overall dietary patterns and clinical cardiovascular events; (b) randomized controlled feeding trials evaluating overall dietary patterns and cardiovascular risk factors (LDL-cholesterol, SBP); and (c) a large RCT evaluating overall dietary patterns and clinical cardiovascular events. For each, we compared the observed effect from the dietary pattern study to the estimated RR calculated by jointly considering the individual etiologic effects (RRs) for each dietary component in that pattern.

For prospective cohorts evaluating overall diet patterns and CVD events,[[2-](#_ENREF_9)6] the observed multivariable-adjusted RR in each category (e.g., quintile) of the dietary pattern was compared to the estimated effect calculated by combining the reported differences in each individual dietary component (e.g., fruits, nuts) across each category of the diet pattern with our estimated individual etiologic effect (RR) for that dietary component, assuming a multiplicative relation between RRs for individual components. We focused on foods and excluded overlapping components (e.g., we included whole grains, fruits, and vegetables; and excluded dietary fiber); we also assumed no benefits from differences in other dietary factors (e.g., coffee) in the pattern for which we had not determined a causal etiologic effect.

For randomized controlled feeding trials of dietary patterns and CVD risk factors, we performed inverse-variance-weighted meta-regression across all of the treatment arms of three large, well-established dietary pattern trials[[7-9](#_ENREF_14)] to estimate the independent effects of five individual dietary components, when consumed as part of an overall dietary pattern, on SBP and LDL-cholesterol. We evaluated achieved changes in fruits, vegetables, nuts, whole grains, and fish simultaneously as independent variables, with changes in SBP or LDL-C as the dependent variable. For each dietary component, we then calculated how the identified change in SBP and LDL-C from the meta-regression would alter cardiovascular risk, based on the established relationship between SBP and LDL-C and clinical events[[10-1](#_ENREF_17)4] assuming independent, multiplicative effects of SBP and LDL-C. These effects, calculated based only on how each dietary component altered SBP and LDL-C in randomized controlled feeding trials of diet patterns, were then compared to our estimated etiologic effect on cardiovascular events for that dietary component. We recognized that the calculated effects based on the feeding trial results might be conservative, as they presume that the summed CVD benefits of these dietary factors are attributable only to effects on SBP and LDL-C, when in reality other pathways of benefit likely exist.

Lastly, we compared the observed vs. estimated risk using findings from the PREDIMED trial, a large RCT evaluating the effects of two overall dietary patterns on CVD incidence.[15] The estimated risk reductions were calculated by combining the observed differences in individual dietary components achieved in the trial with our estimated quantitative effects for each dietary component, assuming multiplicative effects of each individual component.

Each of these validity analyses demonstrated that the estimated etiologic effects for individual dietary components were very similar to what would be expected based on these other lines of evidence.[[1](#_ENREF_8)] **(S11-S13 Table**).

The related question of dietary complements and substitutes was also considered and discussed at length during our study design. In brief, because the diet-disease etiologic effects (relative risks) for the changes in the targeted dietary factors are based on long-term prospective cohort studies, the estimates already incorporate all of the average substitutes and complements across the population. In other words, a cohort study comparing risk associated with higher vs. lower SSB intake already implicitly incorporates the varying dietary substitutes and complements associated with the observed difference in risk, in that people with lower SSB intake may drink more juice or milk, for example. If any of our intervention strategies additionally aimed to encourage or discourage specific complements and substitutes, the efficacy could theoretically be augmented above the currently calculated average effect.

**References**

1. Micha R, Shulkin ML, Penalvo JL, Khatibzadeh S, Singh GM, Rao M, et al. Etiologic effects and optimal intakes of foods and nutrients for risk of cardiovascular diseases and diabetes: Systematic reviews and meta-analyses from the Nutrition and Chronic Diseases Expert Group (NutriCoDE). PLoS One. 2017;12(4):e0175149. Epub 2017/04/28. doi: 10.1371/journal.pone.0175149. PubMed PMID: 28448503; PubMed Central PMCID: PMCPMC5407851.

2. Hu FB, Rimm EB, Stampfer MJ, Ascherio A, Spiegelman D, Willett WC. Prospective study of major dietary patterns and risk of coronary heart disease in men. Am J Clin Nutr. 2000;72(4):912-21. PubMed PMID: 11010931.

3. Fung TT, Willett WC, Stampfer MJ, Manson JE, Hu FB. Dietary patterns and the risk of coronary heart disease in women. Archives of internal medicine. 2001;161(15):1857-62. PubMed PMID: 11493127.

4. Fung TT, Rexrode KM, Mantzoros CS, Manson JE, Willett WC, Hu FB. Mediterranean diet and incidence of and mortality from coronary heart disease and stroke in women. Circulation. 2009;119(8):1093-100. Epub 2009/02/18. doi: 10.1161/circulationaha.108.816736. PubMed PMID: 19221219; PubMed Central PMCID: PMCPMC2724471.

5. Trichopoulou A, Bamia C, Trichopoulos D. Anatomy of health effects of Mediterranean diet: Greek EPIC prospective cohort study. BMJ (Clinical research ed). 2009;338:b2337. Epub 2009/06/25. doi: 10.1136/bmj.b2337. PubMed PMID: 19549997; PubMed Central PMCID: PMCPMC3272659.

6. Martinez-Gonzalez MA, de la Fuente-Arrillaga C, Lopez-Del-Burgo C, Vazquez-Ruiz Z, Benito S, Ruiz-Canela M. Low consumption of fruit and vegetables and risk of chronic disease: a review of the epidemiological evidence and temporal trends among Spanish graduates. Public Health Nutr. 2011;14(12A):2309-15. Epub 2011/12/15. doi: 10.1017/s1368980011002564. PubMed PMID: 22166189.

7. Appel LJ, Moore TJ, Obarzanek E, Vollmer WM, Svetkey LP, Sacks FM, et al. A clinical trial of the effects of dietary patterns on blood pressure. DASH Collaborative Research Group. N Engl J Med. 1997;336(16):1117-24. Epub 1997/04/17. doi: 10.1056/nejm199704173361601. PubMed PMID: 9099655.

8. Sacks FM, Svetkey LP, Vollmer WM, Appel LJ, Bray GA, Harsha D, et al. Effects on blood pressure of reduced dietary sodium and the Dietary Approaches to Stop Hypertension (DASH) diet. DASH-Sodium Collaborative Research Group. N Engl J Med. 2001;344(1):3-10. PubMed PMID: 11136953.

9. Appel LJ, Sacks FM, Carey VJ, Obarzanek E, Swain JF, Miller ER, 3rd, et al. Effects of protein, monounsaturated fat, and carbohydrate intake on blood pressure and serum lipids: results of the OmniHeart randomized trial. JAMA. 2005;294(19):2455-64. PubMed PMID: 16287956.

10. Di Angelantonio E, Sarwar N, Perry P, Kaptoge S, Ray KK, Thompson A, et al. Major lipids, apolipoproteins, and risk of vascular disease. JAMA. 2009;302(18):1993-2000. Epub 2009/11/12. doi: 10.1001/jama.2009.1619. PubMed PMID: 19903920; PubMed Central PMCID: PMCPMC3284229.

11. Baigent C, Keech A, Kearney PM, Blackwell L, Buck G, Pollicino C, et al. Efficacy and safety of cholesterol-lowering treatment: prospective meta-analysis of data from 90,056 participants in 14 randomised trials of statins. Lancet. 2005;366(9493):1267-78. PubMed PMID: 16214597.

12. Law MR, Wald NJ, Rudnicka AR. Quantifying effect of statins on low density lipoprotein cholesterol, ischaemic heart disease, and stroke: systematic review and meta-analysis. BMJ. 2003;326(7404):1423. Epub 2003/06/28. doi: 10.1136/bmj.326.7404.1423. PubMed PMID: 12829554; PubMed Central PMCID: PMCPMC162260.

13. Lawes CM, Rodgers A, Bennett DA, Parag V, Suh I, Ueshima H, et al. Blood pressure and cardiovascular disease in the Asia Pacific region. J Hypertens. 2003;21(4):707-16. Epub 2003/03/27. doi: 10.1097/01.hjh.0000052492.18130.07. PubMed PMID: 12658016.

14. Lewington S, Clarke R, Qizilbash N, Peto R, Collins R, Prospective Studies C. Age-specific relevance of usual blood pressure to vascular mortality: a meta-analysis of individual data for one million adults in 61 prospective studies. Lancet. 2002;360(9349):1903-13. PubMed PMID: 12493255.

15. Estruch R, Ros E, Salas-Salvado J, Covas MI, Corella D, Aros F, et al. Primary prevention of cardiovascular disease with a Mediterranean diet. The New England journal of medicine. 2013;368(14):1279-90. Epub 2013/02/26. doi: 10.1056/NEJMoa1200303. PubMed PMID: 23432189.
